# IGF1R Derived PI3K/AKT Signaling Maintains Growth in a Subset of Human T-Cell Acute Lymphoblastic Leukemias

**DOI:** 10.1371/journal.pone.0161158

**Published:** 2016-08-17

**Authors:** Samuel Gusscott, Catherine E. Jenkins, Sonya H. Lam, Vincenzo Giambra, Michael Pollak, Andrew P. Weng

**Affiliations:** 1 Terry Fox Laboratory, BC Cancer Agency, Vancouver, BC, V5Z 1L3, Canada; 2 Department of Oncology, McGill University, Montreal, Quebec, Canada; Queen's University Belfast, UNITED KINGDOM

## Abstract

Insulin-like growth factor 1 receptor (IGF1R) is a prevalent signaling pathway in human cancer that supports cell growth/survival and thus contributes to aggressive biological behavior. Much work has gone into development of IGF1R inhibitors; however, candidate agents including small molecule tyrosine kinase inhibitors and blocking antibodies have yet to fulfill their promise clinically. Understanding cellular features that define sensitivity versus resistance are important for effective patient selection and anticipation of outgrowth of a resistant clone. We previously identified an important role for IGF signaling in T-cell acute lymphoblastic leukemia (T-ALL) relying primarily upon genetically defined mouse models. We present here an assessment of IGF1R dependence in human T-ALL using a broad panel of 27 established cell lines that capture a spectrum of the genetic variation that might be encountered in clinical practice. We observed that a subset of cell lines are sensitive to IGF1R inhibition and are characterized by high levels of surface IGF1R expression and PTEN positivity. Interestingly, lentiviral expression or knock-down of PTEN in PTEN-negative/positive cell lines, respectively, had limited effects on their response to IGF1R inhibition, suggesting that PTEN contributes to, but does not define IGF dependence. Additionally, we characterize downstream PI3K/AKT signaling as dominant over RAS/RAF/MEK/ERK in mediating growth and/or survival in this context. Finally, we demonstrate that IGF and interleukin-7 (IL-7) fulfill non-overlapping roles in supporting T-ALL growth. These findings are significant in that they reveal cellular features and downstream mechanisms that may determine the response of an individual patient’s tumor to IGF1R inhibitor therapy.

## Introduction

T-cell acute lymphoblastic leukemia (T-ALL) is an aggressive cancer of immature T cells that has been shown to be reliant on multiple signaling pathways to maintain growth and survival. In some instances activation of these pathways is cell autonomous, occurring for instance by mutational activation of an oncogene (e.g. NOTCH1[1]) or loss of a tumor suppressor (e.g. PTEN[2, 3]), while in other instances activation requires stimulation from the environment (e.g. IGF1, IL-7[4, 5]).

The insulin-like growth factor 1 receptor (IGF1R) is a transmembrane receptor tyrosine kinase, closely related to the insulin receptor (InsR), that forms homodimers or heterodimerizes with InsR to recognize its ligands, IGF-1 and IGF-2[6]. Upon ligand binding, IGF1R activates multiple downstream signaling cascades, the two most prominent being PI3K/AKT and RAS/RAF/MEK/ERK. PI3K/AKT enhances cellular metabolism and protein synthesis via mTOR and enhances survival via BAD/Bcl2, p53, NF-κB, and FOXOs, whereas RAS/RAF/MEK/ERK activation generally results in increased cellular proliferation. Early *in vitro* experiments showed IGF1 signaling to be important for neoplastic cell proliferation[7] as well as initial transformation[8] and subsequent *in vivo* experiments re-enforced this important role[9]. In addition correlative population based studies have suggested a link between circulating serum IGF1 levels risk of cancer development for numerous cancer types[6].

Mutations in IGF1R are rare, and none to date have been definitively characterized to activate signaling[10, 11]. On the other hand, mutations activating both canonical downstream signaling pathways, PI3K/AKT and RAS/RAF/MEK/ERK, occur frequently in human cancers and have been implicated in the pathogenesis of T-ALL[12, 13]. As well, we and others have reported previously that IGF1R is upregulated both transcriptionally[4, 14] and post transcriptionally[15] in T-ALL by NOTCH1, a prominent oncogene in the disease[1], and that IGF signaling contributes to growth/survival of bulk cells and also to leukemia-initiating activity[4]. These observations suggest that pharmacologic inhibition of IGF signaling may have a therapeutic role in T-ALL, both in terms of treating bulk disease as well as in targeting leukemia stem cells to prevent relapse.

IGF1R inhibitors have shown efficacy in numerous pre-clinical studies in solid tumors including non-small cell lung cancer, breast cancer, adrenocortical carcinoma, and Ewing sarcoma[16], and also in hematologic malignancies such as myeloma, CLL, B-ALL, T-ALL, and AML[4, 17–20]. Several agents have advanced to clinical trials[21]; however, to date none have been approved for use outside of investigational studies due to limited efficacy and in some instances metabolic toxicity[22]. It has been suggested that efficacy could be improved in selected patient groups with predictive biomarkers and in combination with complementary therapies that target PI3K/AKT and RAS/RAF/MEK/ERK pathways simultaneously[23]. In order to investigate the potential efficacy of IGF signaling inhibitors in human T-ALL, we tested two clinical grade IGF1R inhibitors, a humanized monoclonal blocking antibody, CP-751,871[24], and a small molecule tyrosine kinase inhibitor (TKI) with activity against both IGF1R and InsR, BMS-754807[25], against a broad panel of 27 human T-ALL cell lines. We describe here that a subset of cell lines demonstrates sensitivity to these agents and characterize genetic/phenotypic features that define cellular dependence on IGF signaling.

## Materials and Methods

### Cell culture

All established human T-ALL cell lines were obtained from the laboratories of Drs. Thomas Look (DFCI, Boston), Jon Aster (Brigham & Women’s Hospital, Boston), and Adolfo Ferrando (Columbia University, New York) and have undergone extensive genotypic characterization including STR DNA typing (PowerPlex 16 HS, Promega) [1, 3, 26]. Known gene mutations in PI3K/AKT and MAPK pathways are summarized in S1 Table. Cell lines were grown in RPMI 1640 medium supplemented with 10% FCS, 1 mM sodium pyruvate, 2 mM l-glutamine, and antibiotics. Recombinant human IGF-1, IL-7, and SDF-1α/CXCL12 (Peprotech) were resuspended in PBS/1% BSA prior to addition to culture media. Phorbol 12-myristate 13-acetate (PMA, Sigma P1585) was resuspended in DMSO prior to addition to culture media.

### Drugs

IGF1R inhibitors BMS-754807 and CP-751,871 were obtained under Material Transfer Agreement from their respective manufacturers. The PI3Kγ-selective inhibitor, AS-604850, was obtained from Cayman Chemical (Cat# 10010175). BMS-754807 and AS-604850 were resuspended in DMSO and diluted in PBS or culture media prior to addition to cell cultures. CP-751,871 was diluted in PBS/1% BSA or culture media prior to addition to cell cultures.

### Viable cell assay

Viable cell numbers were determined by the CellTiter-Blue cell viability assay (Promega). Briefly, 2 x 10^4^–3 x 10^4^ cells/100 μl culture media were seeded per well of a 96-well plate, various cytokines/growth factors/inhibitors were added, and cells cultured for 3 days at 37°C in 5% CO_2_. CellTiter Blue reagent was added per manufacturer’s protocol and incubated at 37°C for 30–45 minutes prior to fluorescence measurement at 590 nm using a GENios FL microplate reader (Tecan). All assays were performed in triplicate.

### Viral vectors

All lentiviral expression constructs were generated using a pRRL lentiviral backbone with MNDU3 promoter and PGK-GFP or PGK-NGFR marker as described[27]. The constitutively active KRAS(G12D) mutant was cloned from the murine T-ALL cell line 144CLP[28]. A myristoylated, constitutively active form of Akt1 was cloned from pUSEmyrAkt1 (Upstate). A constitutively active form of IGF1R, CD8-IGF1R, was created by fusing the extracellular/transmembrane domain of human CD8a (amino acids 1 to 218) with the intracellular beta chain of human IGF1R (amino acids 964 to 1367) as described[29]. The constitutively active IL7Raα (p.L242_L243insLSRC) mutant was cloned from the human T-ALL cell line DND41 as described[30]. PTEN was cloned from a wild-type patient T-ALL sample.

Lentiviral shRNA knockdown constructs targeting PTEN were identified from the RNAi Consortium (TRCN0000002746 and TRCN0000002749) and cloned into pLKO.1-GFP[27]. The non-silencing scramble shRNA was a gift from David Sabatini (Addgene plasmid # 1864). All constructs were verified by sequencing.

### Viral transduction

High titer, replication defective lentivirus was produced utilizing pCMVdR8.74, pCMV-VSV-G, and pRSV-Rev packaging vectors by transient transfection of 293T producer cells, where necessary virus was concentrated using PEG-8000. Viral transduction was performed by spinoculation with 4 μg/ml polybrene as described[15].

### Western blot

Whole cell protein extracts were prepared using RIPA buffer (50 mM Tris-HCl pH 8, 150 mM NaCl, 1% NP-40, 0.25% Na-deoxycholate, 1 mM EDTA) supplemented with 1 mM NaF, 1 mM Na_3_VO_4_, 2.5 mM Na-pyrophosphate, 1 mM phenyl-methylsulfonyl-fluoride and 1x Protease Inhibitor Cocktail Set III (Calbiochem). Lysates were cleared by centrifugation at 14,000 × *g* for 10 minutes at 4°C, separated by SDS-PAGE, and transferred to PVDF membranes. Blots were probed with antibodies directed against PTEN (Y184, Abcam), ZAP70 (99F2, Cell Signaling Technology), AKT (#9272, Cell Signaling Technology), phospho-ERK1/2 (Thr202/Tyr204) (E10, Cell Signaling Technology), ERK2 (C-14, Santa Cruz Biotechnology), RAS(G12D) (D8H7, Cell Signaling Technology), IGF1Rβ (C-20, Santa Cruz Biotechnology), and β-actin (AC-15, Sigma) followed by HRP-conjugated secondary antibody and chemiluminescence detection (Pierce). Band intensities were quantitated using Image Studio Lite (LI-COR) or ImageJ (NIH) software.

### Ligand stimulation assay

Cells were serum starved by culturing in serum-free media for ~24 hours, then stimulated by addition of recombinant ligand or 10% fetal bovine serum. Cells were fixed 10 minutes later by addition of methanol-free paraformaldehyde (Electron Microscopy Services, Cat #15710) to 1.5% final concentration, then permeabilized with ice-cold methanol for at least 30 minutes before analysis by flow cytometry.

### Flow cytometry

Cell surface expression levels of IL7Rα and IGF1R were determined by staining fresh cells with antibodies against IL7Rα (clone A019D5; Cat# 351317, Biolegend) or IGF1R (clone αIR3; Cat# MABS192, EMD Millipore), respectively. The latter was detected by staining with an APC-conjugated goat anti-mouse secondary antibody (BioLegend, Cat# 405308). Intracellular phospho-protein analysis was performed by staining formaldehyde fixed, methanol permeabilized cells with AlexaFluor647-conjugated antibodies against phospho-AKT (Ser473) (clone D9E; Cat# 4075, Cell Signaling), phospho-ERK1/2 (Thr202/Tyr204) (clone D13.14.4E; Cat# 4284, Cell Signaling), phospho-STAT5 (Y694) (clone 47; Cat# 612599, BD Biosciences), or isotype control (clone DA1E; Cat# 2985, Cell Signaling). Data was acquired on FACSCalibur or LSRFortessa cytometers (Becton Dickinson) and analyzed using FlowJo software (Tree Star).

## Results

### Efficacy of pharmacologic IGF1R inhibitors in human T-ALL cell lines

Our prior work has revealed that IGF signaling is important in T-ALL; however, we addressed this issue by relying most heavily upon genetically defined mouse models where we were able to exclude the contribution of other genetic variables. In fact, the spectrum of human T-ALL presents several common genetic alterations that may reasonably be expected to modulate the IGF signaling pathway, and in this sense, may potentially limit the clinical efficacy of IGF inhibitors. To begin to address this issue, we screened two clinically relevant IGF inhibitors, CP-751,871 (figitumumab), an IGF1R blocking antibody, and BMS-754807, a small molecule dual IGF1R/InsR tyrosine kinase inhibitor, against a broad panel of 27 human T-ALL cell lines and scored for effects on overall cell growth/survival. We used CP-751,871 at a dose of 1 μg/ml which others have reported to be sufficient to maximally block IGF-1 binding and ~4-fold greater than necessary to maximally block IGF1R autophosphorylation[24]. As well, we observed maximal growth inhibition of selected T-ALL cell lines to be achieved at the 1 μg/ml dose, with no additional effect at doses up to 100 μg/ml (S1 Fig). We used BMS-754807 at a dose of 0.5 μM based on our own results (data not shown) and those of others which support that whereas the EC50 for IGF1R-specific effects are in the 0.1 μM range, IGF1R non-specific effects arise at doses ≥1 μM[31].

**Fig 1:**
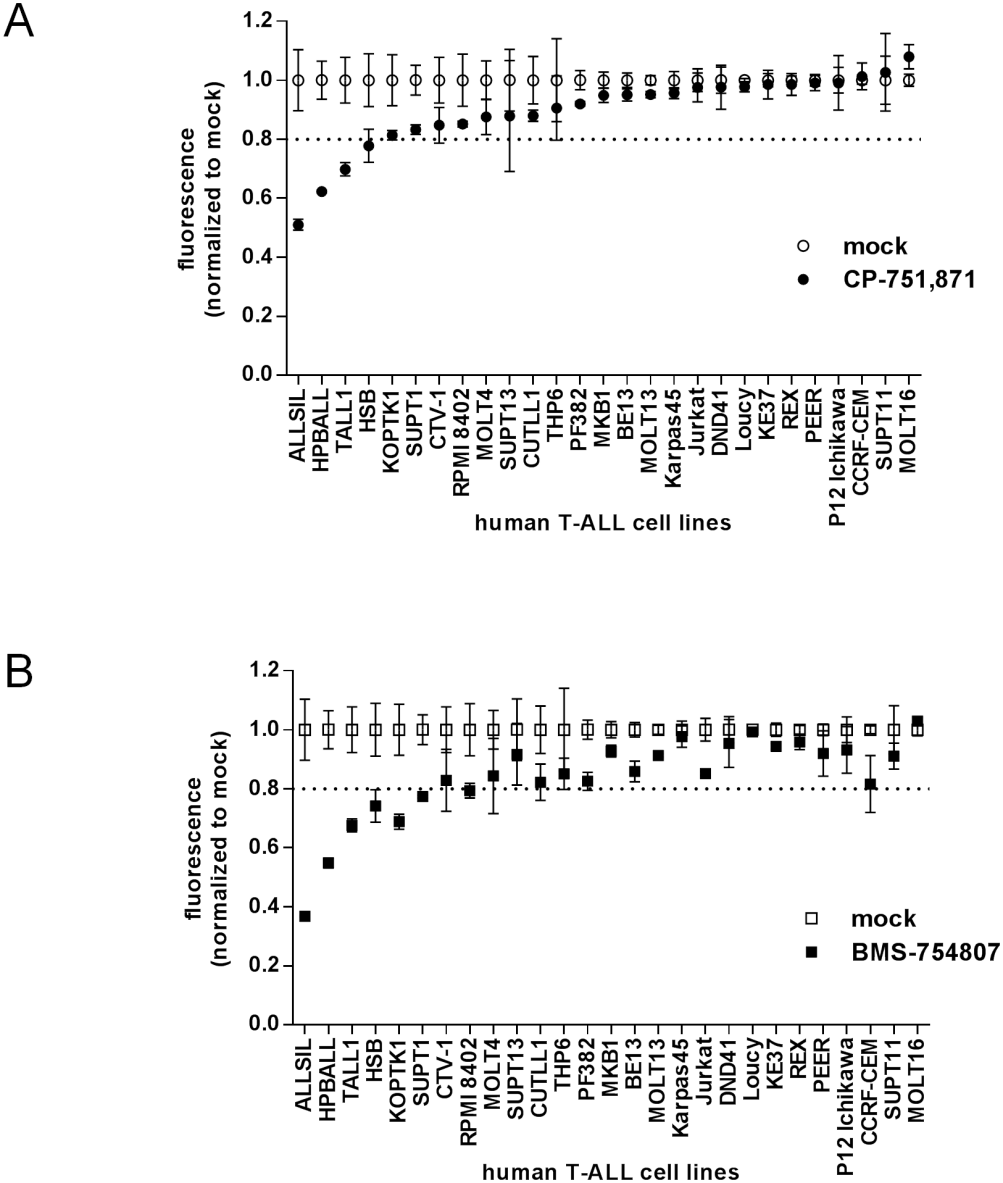
Pharmacological inhibition of IGF1R restricts growth of a subset of human T-ALL cell lines. Cell growth as measured by resazurin reduction assay. Twenty-seven human T-ALL cell lines were cultured *in vitro* for 3 days with either (**A**) IGF1R blocking antibody (CP-751,871; 1 μg/ml) versus PBS vehicle control (mock) or (**B**) dual IGF1R/InsR tyrosine kinase inhibitor (BMS-754807; 0.5 μM) versus DMSO vehicle control (mock). Mean resorufin (reduced resazurin) fluorescence values +/- SD after normalization to mock-treated controls are plotted for assays performed in triplicate. Cell lines are rank ordered left-to-right by decreasing effect of the CP-751,871 blocking antibody. The horizontal dotted line indicates the 20% growth inhibition level.

One-third of cell lines showed a statistically significant effect of CP-751,871 (9/27 with p<0.05 difference between mock and treated by t-test; median 19% inhibition, range 5–49%,) including 4/27 exhibiting more than 20% decrease in cell growth (Fig 1A), while over half were affected by BMS-754807 (15/27 with p<0.05 difference between mock and treated by t-test; median 18% inhibition, range 6–63%) including 7/27 exhibiting more than 20% decrease in growth (Fig 1B). There was strong correlation in response to CP-751,871 and BMS-754807 among these cell lines (Pearson r = 0.932, p<0.0001); however, there was also a subset of cell lines which were more responsive to BMS-754807 than CP-751,807 (7/27 with statistically significant difference of greater than 10%; S2 Fig), potentially reflecting the contribution of InsR or other related tyrosine kinases. As well, it is clear that many cell lines are indeed resistant to IGF1R inhibition[31], and in fact these agents have not shown great success in clinical trials[32, 33]. Accordingly, we sought to understand potential mechanisms that underlie resistance to IGF1R inhibition.

### Effect of IGF1R expression level

**Fig 2:**
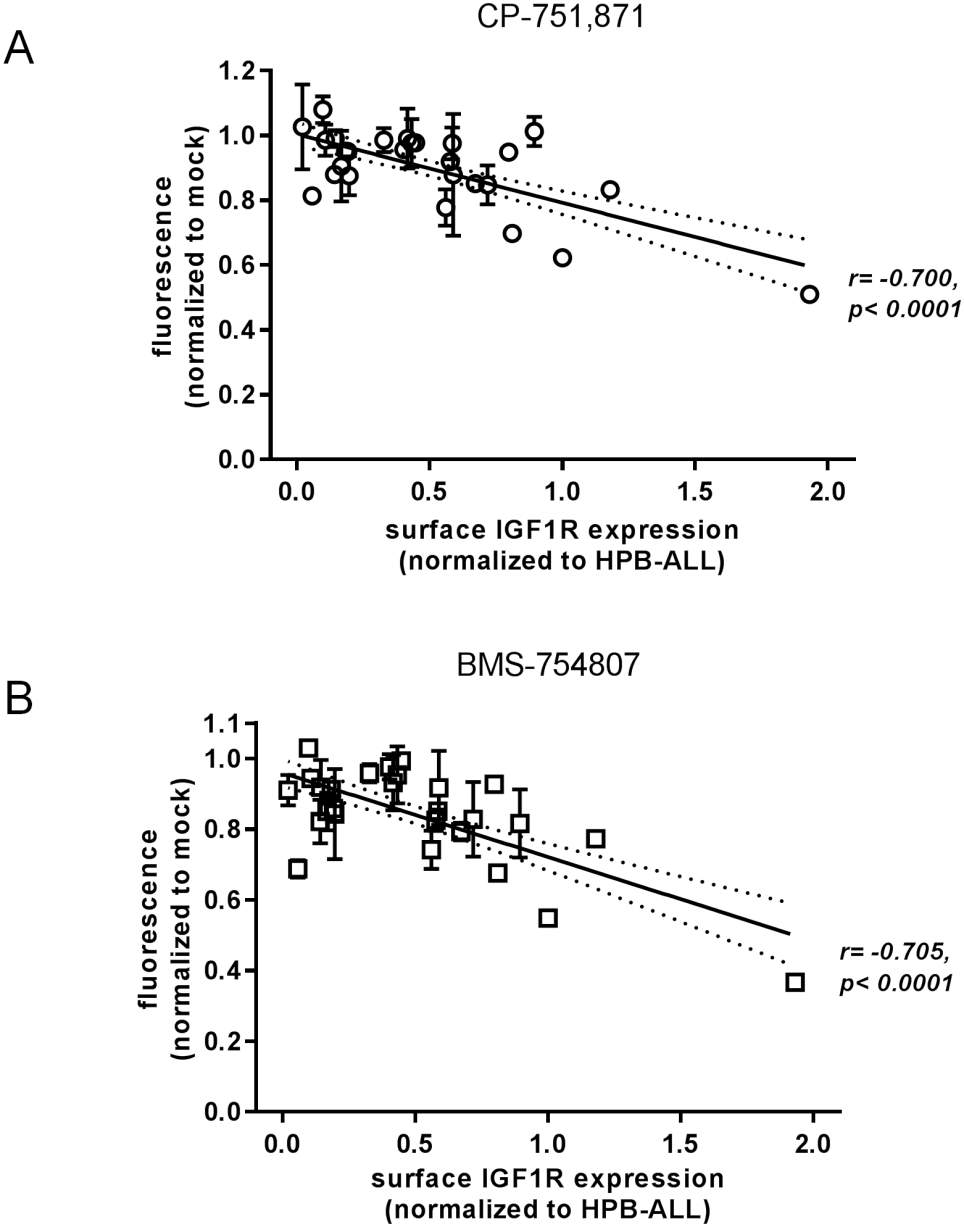
Sensitivity to IGF1R inhibition correlates with surface IGF1R expression level. Plots of cell growth (normalized resorufin fluorescence data from Fig 1) vs. surface IGF1R expression level (mean fluorescence intensity by flow cytometry from S3 Fig). Linear regression lines are depicted with the 95% confidence interval indicated by flanking dotted lines. Pearson correlation r values and associated significance p-values are as indicated.

One obvious variable that might be expected to affect a cell’s response to IGF inhibition would be the level of IGF1R expressed on the cell surface. Indeed, we found the surface IGF1R level (S3 Fig) to be inversely correlated with cell growth under inhibition with both CP-751,871 (Pearson r = −0.700, p<0.0001) (Fig 2A) and BMS-754807 (Pearson r = −0.705, p<0.0001) (Fig 2B) such that cells with higher levels of surface IGF1R expression were more sensitive to IGF1R inhibition. Of note, this correlation is driven largely by those cell lines with the highest levels of IGF1R expression such that if the top 3 IGF1R-expressing cell lines are excluded from the analysis, the correlation loses significance (S4 Fig). Nonetheless, this relation may suggest that cells which gain growth/survival advantage from IGF signaling have been selected to upregulate expression of IGF1R on the cell surface and thus maximize their ability to respond to ambient levels of IGF factors in the surrounding environment. Importantly, we confirmed that IGF1R expressed on the surface of T-ALL cell lines is indeed responsive to stimulation by IGF1 ligand as measured by activation of AKT (S5 Fig). Thus, high levels of IGF1R expression on the cell surface may be taken as a feature which would suggest a given tumor is likely to respond to inhibition of IGF signaling.

### Downstream effector pathways

**Fig 3:**
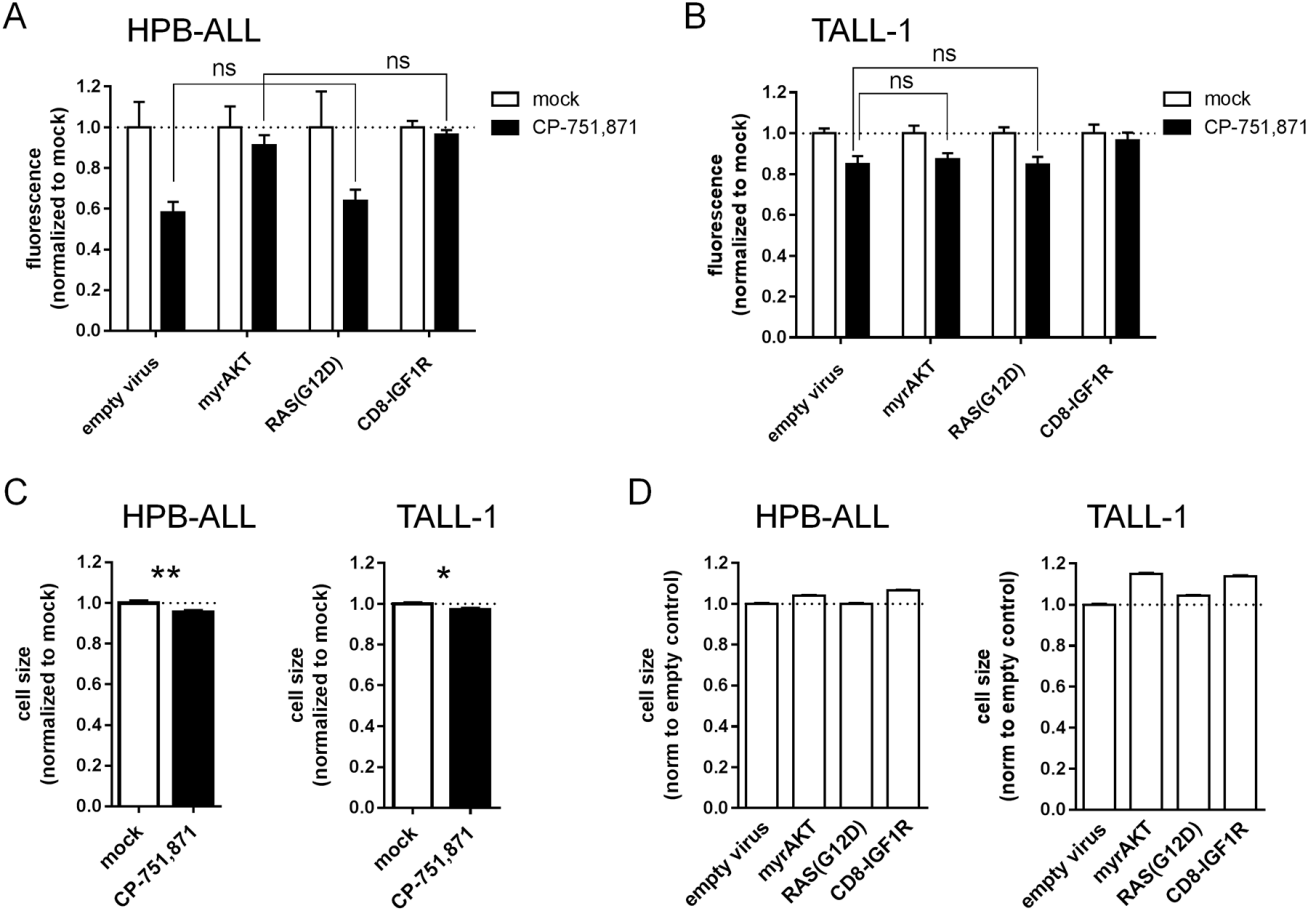
Constitutive activation of AKT, but not RAS rescues T-ALL cells from IGF1R inhibition. (**A,B**) Cell growth as measured by resazurin reduction. T-ALL cells were transduced with lentiviral vectors as indicated, FACS sorted, and then cultured *in vitro* with IGF1R blocking antibody (1 μg/ml CP-751,871) for 3 days. Mean resorufin fluorescence values +/- SD after normalization to respective mock-treated controls are plotted for assays performed in triplicate. *ns*, *not significant (2-way ANOVA with Sidak’s multiple comparisons test)*. (**C,D**) Cell size as measured by forward light scatter. (**C**) T-ALL cells were treated with CP-751,871 (1 μg/ml) for 3 days. Data are depicted for gated live events. (**D**) T-ALL cells were transduced with lentiviral vectors as indicated. Data are depicted for gated live GFP+ events. Mean forward light scatter values +/- 95% confidence interval are plotted after normalization to mock-treated control cells (**C**) or to untransduced cells in the same culture, then scaled to the empty virus control (**D**). *, *p<0*.05; **, *p<0*.*01 (Student’s t-test)*.

In our prior study examining the contribution of IGF1R to leukemia propagation *in vivo*, we found that a hypomorphic allele of IGF1R (IGF1R^neo^) abrogated serial transplantability of mouse T-ALL[4]. As might be expected in any biological system, we did observe a limited number of clones to bypass the requirement for high-level IGF1R expression and in doing so restored transplantability. In a sense, the IGF1R^neo^ mouse model may be predictive of what might be expected to occur in T-ALL patients following IGF1R inhibitor therapy. To explore this issue specifically in the context of human T-ALL, we elected to test prospectively the ability of candidate downstream signaling elements to render previously sensitive cell lines resistant to IGF1R inhibition. Based on the accumulated literature supporting that IGF signals bifurcate into two major arms, PI3K/AKT and RAS/RAF/MEK/ERK, we first tested whether constitutive activation forms of either or both of these would restore cell growth following treatment with CP-751,871/BMS-754807. Interestingly, a constitutively active myristoylated AKT (myrAKT) construct rescued HPB-ALL cells from CP-751,871-induced growth inhibition to an extent on par with a constitutively active CD8-IGF1R fusion protein[29] which we employed as a positive control in this assay (Fig 3A and S6 Fig). The CD8-IGF1R fusion combines the extracellular/transmembrane domain of human CD8 with the intracellular IGF1Rβ chain[29] and thus achieves constitutive activation of IGF1R signaling by homodimerization of the chimeric receptor mediated by the CD8 moiety[29], but yet is not targeted by the CP-751,871 blocking antibody. To confirm the specificity of the constitutively active CD8-IGF1R positive control, we demonstrated in the same assay that Y950F or K1003A point mutants of the CD8-IGF1R construct which are unable to interact with downstream effectors IRS1/2 and SHC, or lack kinase activity, respectively, were unable to rescue CP-751,871-induced growth inhibition (S7 Fig).

In contrast to myrAKT, the constitutively active RAS(G12D) mutant showed little to no evidence of rescue from CP-751,871-induced growth inhibition in HPB-ALL cells and performed similarly to empty virus which we employed as a negative control in this assay (Fig 3A and S6 Fig). In fact, RAS(G12D) failed to induce downstream ERK1/2 phosphorylation in 3 different T-ALL cell lines as measured by either western blot or flow cytometry assay (S8A and S8B Fig), implying that the RAS/RAF/MEK/ERK pathway is not readily activated in these contexts. Importantly, the signaling pathway is intact, at least in HPB-ALL cells from RAF onwards, as evidenced by induction of phospho-ERK1/2 by the phorbol ester PMA which acts via PKC to RAF[34] (S8C Fig), and the RAS(G12D) construct itself functions properly as shown by induction of phospho-ERK1/2 in 293T cells (S9 Fig). Nonetheless, the level of RAS(G12D) protein expression obtained was modest (S6 Fig), and thus the question remains open as to whether more potent stimulation of RAS signaling than can be obtained with the G12D construct is capable of activating ERK in these contexts.

We attempted to confirm these results in an additional CP-751,871-sensitive cell line, TALL-1, but did not find myrAKT to be effective in restoring growth (Fig 3B and S6 Fig). We did note a modest, but nonetheless statistically significant decrease in cell size for both HPB-ALL and TALL-1 after treatment with CP-751,871 (Fig 3C), and that myrAKT induced an increase in cell size which was not seen as robustly with RAS(G12D) (Fig 3D and S6 Fig). We corroborated these results with intracellular phospho-AKT levels which revealed that whereas treatment with CP-751,871 resulted in decreased steady-state phospho-AKT levels, both myrAKT and CD8-IGF1R increased phospho-AKT levels while RAS(G12D) did not (S10 Fig). Of note, the TALL-1 cell line already carries an endogenous NRAS(G12D) mutation (S1 Table) which we confirmed was expressed at the protein level by western blot (S6 Fig). The lack of detectable pERK in these cells under steady-state conditions (S8 Fig) raises the possibility that activated RAS may signal through downstream pathways other than MAPK/ERK in this situation. Unfortunately, we were unable to score these constructs in the most sensitive cell line, ALL-SIL, due to its low transduction efficiency (data not shown).

As an alternative to attempting to rescue the effects of IGF1R inhibition with downstream signaling elements, we also asked more directly the extent to which PI3K/AKT and MAPK/ERK signaling cascades were activated downstream of IGF1R in these cells. To this end, we performed phospho-flow analysis for pAKT and pERK in the four most sensitive cell lines (ALL-SIL, HPB-ALL, TALL-1, and HSB) following serum starvation and pulsing with recombinant IGF1 ligand. There was clear and consistent induction of pAKT by IGF1 in all 4 cell lines, but little if any evidence of pERK response to IGF1 (S11 Fig). Taken together, these results support the notion that IGF1R-dependent cell growth phenotypes in a subset of T-ALL are mediated by activation of PI3K/AKT more so than MAPK/ERK signaling. Of note, the effects of myrAKT on cell size, which are presumably operative during G1 phase of the cell cycle via activation of mTOR/S6K1/4EBP1[35], do not translate consistently to cell proliferation, suggesting that in some cellular contexts (e.g. TALL-1) additional signals are required to drive cells across the G1/S checkpoint, but which are presumably provided by other pathways downstream of IGF1R.

### Effect of PTEN

**Fig 4:**
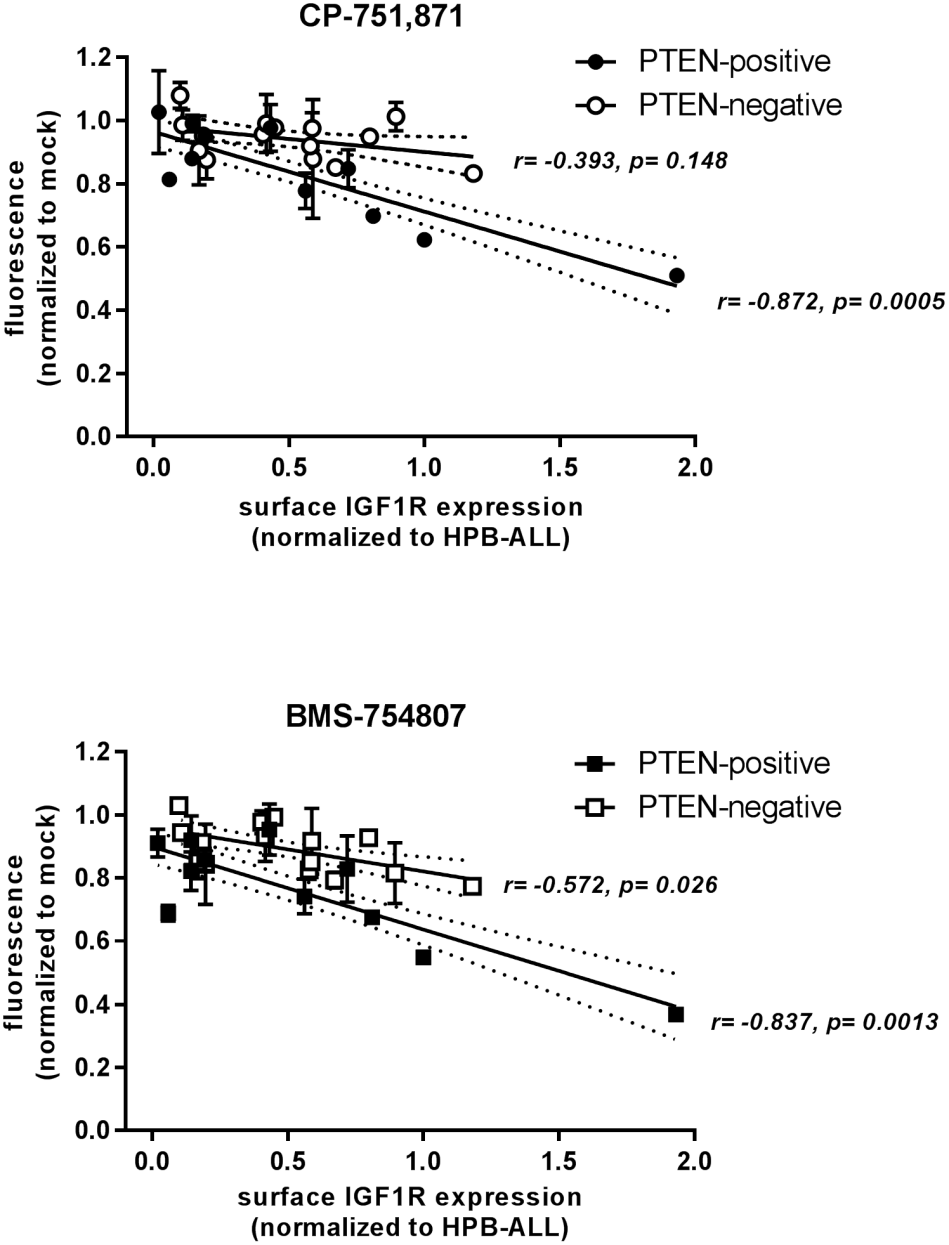
Correlation between IGF1R inhibitor efficacy and surface IGF1R expression level holds up in PTEN-positive cell lines, but less so in PTEN-negative cell lines. Linear regression lines are shown separately for PTEN-positive and -negative subsets, with 95% confidence intervals indicated by flanking dotted lines. Pearson correlation r values and associated significance p-values are indicated for each subset. *Plotted data are identical to those presented in Fig 2, but now segregated into PTEN-positive and -negative subsets*.

Canonical activation of AKT downstream of receptor tyrosine kinases such as IGF1R occurs via PI3K-dependent conversion of PI(3,4)P_2_ to PI(3,4,5)P_3_ at the plasma membrane. The lipid phosphatase PTEN dephosphorylates PIP_3_, converting it back to PIP_2_, and thereby down modulating signaling through AKT. Thus, another major variable that might be expected to modulate a cell’s sensitivity to IGF inhibition would be its PTEN status. Of note, PTEN is deleted or mutated in approximately 10–25% of patient T-ALLs[2, 3, 12], with greater incidence at relapse. Among the 26 cell lines for which PTEN status was available, 11 were positive for PTEN including the 5 most sensitive cell lines (S2 Table and S12 Fig). In fact, there was a significant correlation between PTEN positivity and sensitivity to both CP-751,871 and BMS-754807 (p = 0.007 and 0.004, respectively; one-tailed point-biserial correlation), but this correlation was not absolute as there were indeed occasional examples of PTEN-positive resistant lines (e.g. DND41) and PTEN-negative sensitive lines (e.g. SUPT1) (Fig 1). Of note, there was no significant difference in surface IGF1R expression level between the PTEN-positive and -negative subsets (S13 Fig). Also, the correlation between IGF1R inhibitor efficacy and surface IGF1R expression noted above continues to hold true in the PTEN-positive subset of cell lines (n = 11; Pearson r for CP-751,871 = −0.872, p = 0.0005; Pearson r for BMS-754807 = −0.837, p = 0.0013); however, within the PTEN-negative subset (n = 15), the correlation holds for BMS-754807 (Pearson r = −0.572, p = 0.026), but loses significance for CP-751,871 (Pearson r = −0.393, p = 0.148) (Fig 4). Again, if the top 3 IGF1R-expressing cell lines are excluded from this analysis, all correlations lose statistical significance; however, the PTEN-positive subset shows a positive trend with CP-751,871 that is of borderline significance (p = 0.053; S14 Fig).

**Fig 5:**
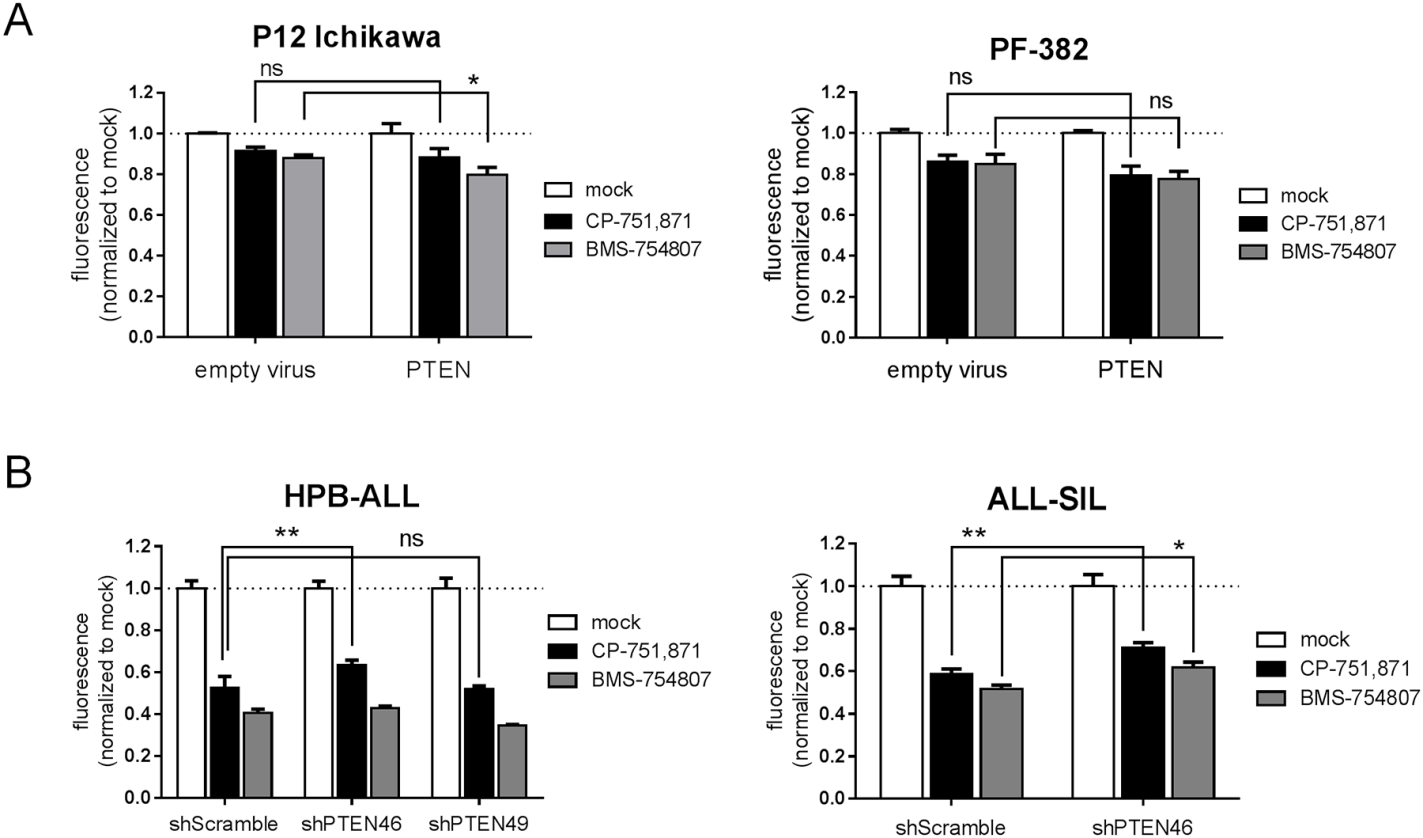
PTEN contributes to, but does not define IGF dependence. Cell growth as measured by resazurin reduction assay. (**A**) PTEN-negative cell lines (P12 Ichikawa and PF-382) or (**B**) PTEN-positive cell lines (HPB-ALL and ALL-SIL) were transduced with lentiviral PTEN expression or knock-down constructs, respectively, FACS sorted, and then cultured *in vitro* with IGF1R blocking antibody (1 μg/ml CP-751,871) or dual IGF1R/InsR tyrosine kinase inhibitor (0.5 μM BMS-754807) for 2–3 days. Mean resorufin fluorescence values +/- SD after normalization to respective mock-treated controls are plotted for assays performed in triplicate. *, *p<0*.05; **, *p<0*.*01; ns*, *not significant (2-way ANOVA with Sidak’s multiple comparisons test*).

To test the association between PTEN and IGF dependence in a more direct manner, we generated isogenic cell lines with and without PTEN by 1) re-expressing PTEN in PTEN-negative cell lines and 2) knocking down PTEN in PTEN-positive cell lines, and then reassessed their response to IGF inhibition. Transduction of PTEN-negative P12 Ichikawa and PF382 cells with PTEN lentivirus (S15A Fig) only mildly enhanced growth inhibition by CP-751,871 or BMS-754807 which was often statistically insignificant (Fig 5A). As well, knock-down of PTEN by 70–90% using lentiviral shRNAs (S15B Fig) only mildly increased resistance to IGF1R inhibition (Fig 5B). From these results, we conclude that the limited effect of enforcing or knocking-down PTEN expression suggests that other, co-occurring genetic variables, perhaps including mutations in other PI3K/AKT pathway elements as have been previously reported in patient T-ALL samples[12], likely function in combination with PTEN loss to confer IGF independence. Of note, it has been reported that PTEN is frequently inactivated by post-translational phosphorylation/oxidation in T-ALL[36]; however, we confirmed that the exogenous, lentivirally expressed PTEN was indeed functional as evidenced by decreased steady-state levels of phospho-AKT (S16 Fig).

### Role of PI3K isoforms

**Fig 6:**
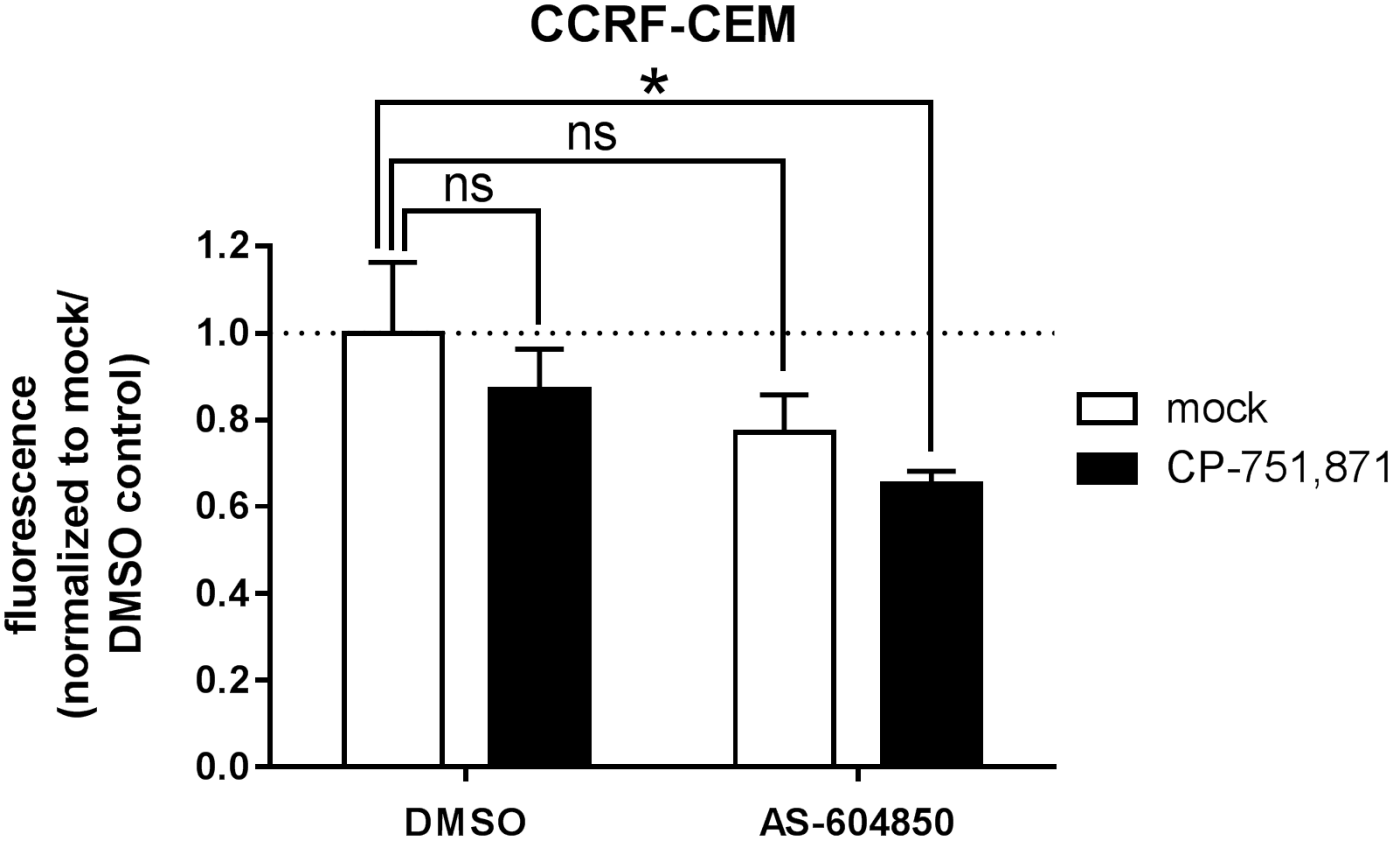
Combined inhibition of IGF1R and PI3Kγ does not block growth of PTEN negative CCRF-CEM cells. Cell growth as measured by resazurin reduction assay. CCRF-CEM cells were cultured *in vitro* with IGF1R blocking antibody (1 μg/ml CP-751,871) with or without a selective PI3Kγ inhibitor (50 μM AS-604850) for 3 days. Mean resorufin fluorescence values +/- SD after normalization to the mock/DMSO control are plotted for assays performed in triplicate. *, *p<0*.*05; ns*, *not significant (2-way ANOVA with Sidak’s multiple comparisons test*).

PI3Ks are composed of a catalytic and a regulatory subunit, the former existing in one of four distinct isoforms, p110α-δ[37]. Tyrosine kinases, such as IGF1R, generally act upon Class IA PI3Ks p110α/β/δ, whereas G-protein coupled receptors (GPCRs) generally act via either the sole Class IB PI3K, p110γ, or p110β [38, 39]. Interestingly, it has been shown that the PTEN-deficient CCRF-CEM T-ALL cell line is reliant upon either p110γ or p110δ such that treatment with a dual specificity inhibitor induces growth arrest and apoptosis[40]. Since IGF1R has been reported to signal through p110α/β/δ in other cellular contexts[41, 42], we wondered whether resistance to IGF1R inhibition might be mediated by p110γ, possibly via an as-yet-uncharacterized GPCR. We tested this hypothesis again using the CEM cell line as it was the only one reported in the aforementioned study that was sensitive to p110γ/δ inhibition and which we found to be resistant to IGF1R inhibition in our screens (Fig 1). We treated these cells with a p110γ-specific inhibitor, AS-604850, at dosing appropriate for *in vitro* cell-based assays[43], both alone and in combination with CP-751,871 antibody on the premise that AS-604850 would block p110γ and CP-751,871 would block IGF1R-dependent p110α/β/δ. We anticipated that the combination might phenocopy the effect seen with dual p110γ/δ[40] or pan-PI3K inhibition[44]. While the combination did indeed have a significant effect on growth (p = 0.01; 2-way ANOVA with Sidak’s multiple comparisons test), there was no significant interaction between them (interaction p = 0.9, 0.03% of total variation; 2-way ANOVA) (Fig 6). We interpret these findings to suggest that there may indeed be a component of p110γ activation in CEM cells, but also that there are likely signaling inputs upstream of p110α/β/δ other than IGF1R. These observations are limited to a single cell line, however, and may not be generalizable. Moreover, the relative contributions of individual p110 isoforms may well vary from tumor to tumor, and possibly as well between subclones within an individual tumor[39]. Although others have observed that pan-inhibition of all four isoforms achieves maximal growth suppression of PTEN-deficient T-ALL cell lines including CEM[44], consideration of side effect profiles will ultimately dictate the choice between pan- and selective isoform inhibitors in the clinic[45].

### Non-overlapping roles of IGF-1 and IL-7

**Fig 7:**
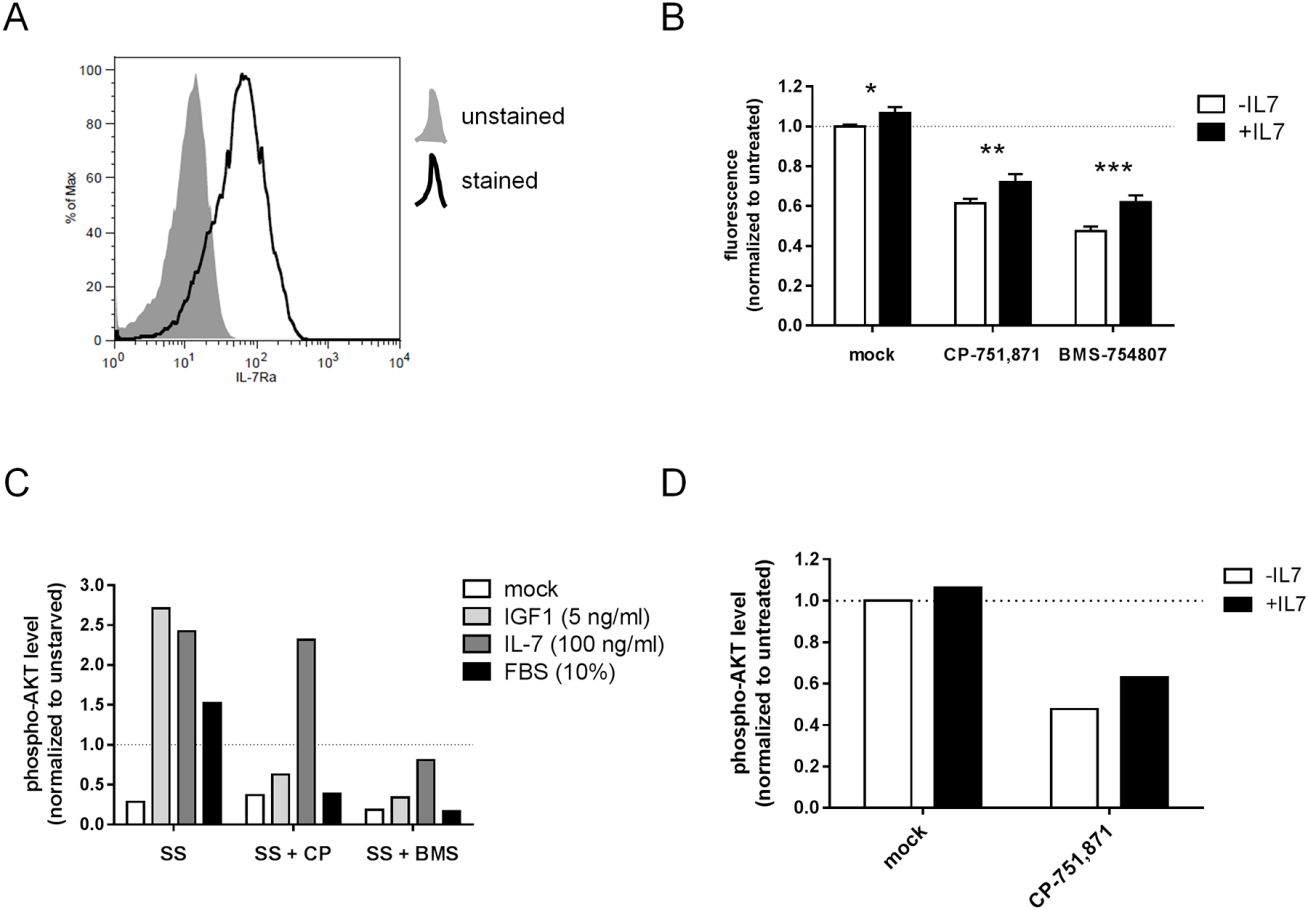
Signaling through IL7R does not rescue T-ALL cells from IGF1R inhibition nor maintain prolonged activation of AKT. (**A**) Flow cytometric analysis for surface IL7R expression level in HPB-ALL cells cultured under routine conditions. (**B**) Cell growth as measured by resazurin reduction assay. HPB-ALL cells were cultured *in vitro* for 3 days with IGF1R blocking antibody (1 μg/ml CP-751,871) or IGF1R/InsR tyrosine kinase inhibitor (0.5 μM BMS-754807) with or without supplemental recombinant IL-7 (100 ng/ml, added once at the beginning of the culture period). Mean resorufin fluorescence values +/- SD after normalization to untreated control are plotted for assays performed in triplicate. *, *p<0*.05; **, *p<0*.01; ***, *p<0*.*001 (2-way ANOVA with Sidak’s multiple comparisons test*). (**C,D**) Flow cytometric analysis for intracellular phospho-AKT. (**C**) HPB-ALL cells were serum starved with or without IGF1R inhibition for 24 hours, and then pulsed with IL-7, IGF-1 or FBS for 10 minutes. Mean fluorescence intensity after normalization to unstarved, untreated control is plotted for a representative example of assays performed in duplicate. *SS*, *serum starved; CP*, *CP-751*, *871 (1 μg/ml); BMS*, *BMS-754807 (0*.*5 μM)*. *(***D**) HPB-ALL cells were cultured *in vitro* for 3 days with IGF1R blocking antibody (1 μg/ml CP-751,871) with or without supplemental recombinant IL-7 (100 ng/ml). Mean fluorescence intensity after normalization to untreated control is plotted for a representative example of assays performed in duplicate.

Prior studies on cytokine/growth factor-dependence in human T-ALL have suggested that IL-7 is a prominent contributor in supporting T-ALL cell growth both *in vitro*[46] and *in vivo*[5], and that this effect is mediated in part through PI3K/Akt[47]. As well, activating mutations in IL7R occur in 10% of pediatric T-ALL cases[48, 49], and transduction of mouse thymocyte progenitors with patient-derived IL7R mutants develop immature T-cell leukemias resembling human ETP T-ALL[50]. Of note, the only cell line in our panel known to harbor an activating IL7R mutation, DND41, is resistant to both CP-751,871 and BMS-754807 inhibitors, yet expresses moderate levels of IGF1R on the surface and is PTEN wild-type, raising the intriguing possibility that constitutive activation of IL7R may stimulate PI3K/AKT sufficiently such that input from IGF1R is not required to maintain cell growth/survival. To determine whether there is indeed overlap between IL-7 and IGF signaling with respect to PI3K/AKT activation in human T-ALL cells, we attempted to rescue CP/BMS-induced growth suppression by supplementing culture media with recombinant IL-7. We selected HPB-ALL as a model for this purpose because it is one of the few cell lines that stably express IL7R on the cell surface (Fig 7A and data not shown). While supplemental IL-7 had a variable, but positive effect on cell growth overall, it did not reverse growth inhibition by either CP-751,871 or BMS-754807 (Fig 7B and S17 Fig). Interestingly, we found that AKT nonetheless underwent phosphorylation following acute stimulation with recombinant IL-7 to an extent comparable to recombinant IGF-1 (Fig 7C).

To address the apparent discrepancy between robust acute activation of AKT by IL-7 in CP-treated cells and the lack of restoration of cell growth, we examined steady-state phospho-AKT levels after 3 days of culture in supplemental IL-7 and surprisingly found only limited enhancement compared to non-supplemented control (Fig 7D). Of note, treatment with CP antibody did effectively reduce levels of phospho-AKT, correlating with its growth inhibitory effect. Importantly, specificity of the CP-751,871 antibody in this context was confirmed by its inhibition of AKT phosphorylation by IGF, but not by IL-7 (Fig 7C), and its lack of effect on IL-7 induced STAT5 phosphorylation (S18A Fig). We observed no effect of either IGF1 or IL-7 on ERK1/2 phosphorylation in this context (S8C and S18B Figs). In contrast to CP-751,871, we noted that BMS-754807 suppressed activation of AKT by both IGF1 and IL-7, indicating a degree of cross-reactivity on IL7R/JAK at the 0.5 μM dose (Fig 7C). It was also notable that CP-751,871 completely inhibited AKT phosphorylation by FBS, suggesting that the majority of AKT stimulating activity in standard FBS-containing culture media is mediated via IGF1R and/or IGF1R/InsR hybrids.

**Fig 8:**
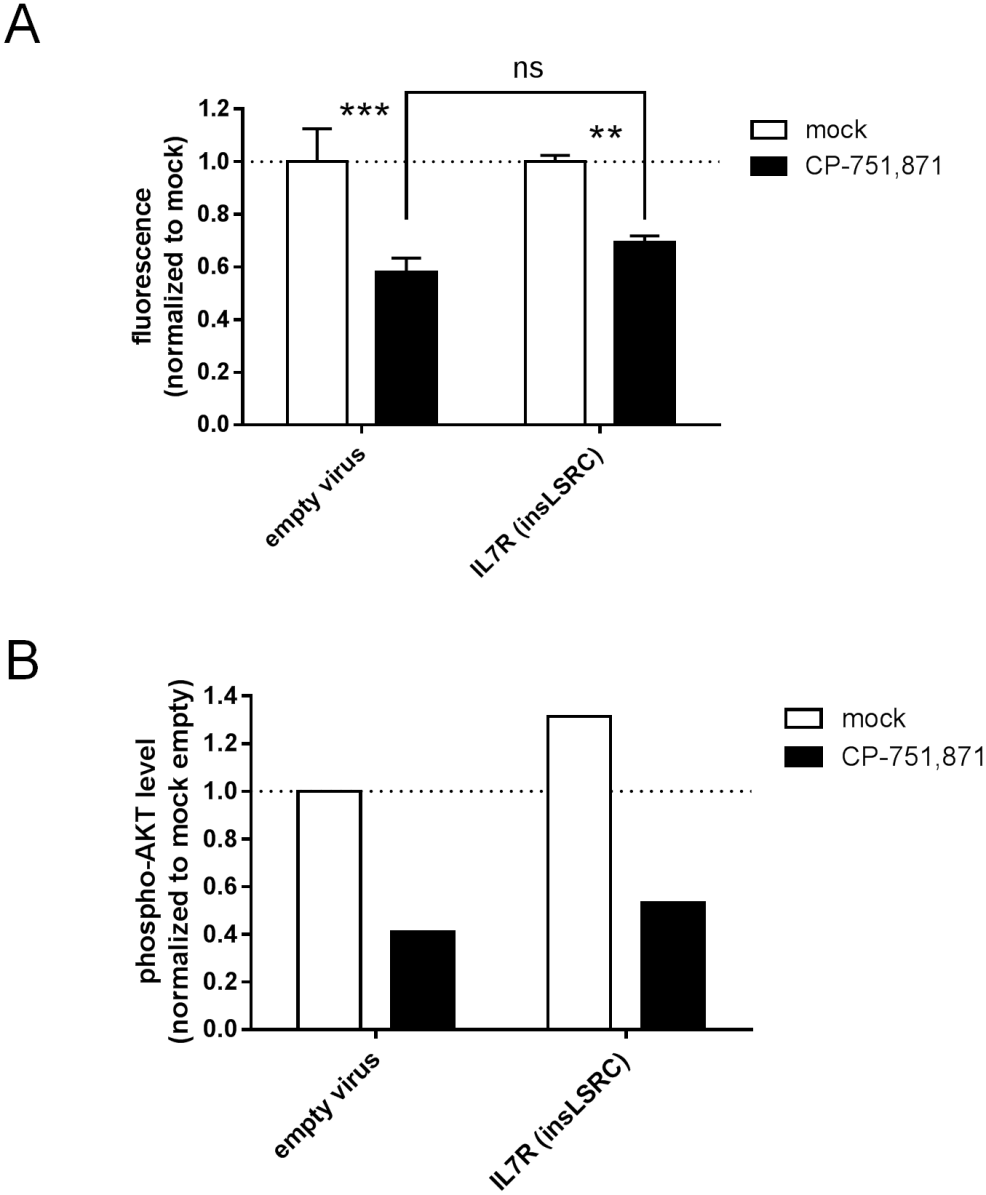
Constitutive activation of IL7R does not confer resistance to IGF1R inhibition. HPB-ALL cells were transduced with a constitutively active IL-7R lentivirus bearing the p.L242_L243 insLSRC mutation, FACS sorted, and cultured *in vitro* with IGF1R blocking antibody (1 μg/ml CP-751,871) for 3 days. (**A**) Cell growth as measured by resazurin reduction assay. Mean resorufin fluorescence values +/- SD after normalization to respective mock-treated controls are plotted for assays performed in triplicate. **, *p<0*.01; ***, *p<0*.*001; ns*, *not significant (2-way ANOVA with Sidak’s multiple comparisons test*). (**B**) Flow cytometric analysis for intracellular phospho-AKT (S473) level. Mean fluorescence intensity after normalization to mock-treated, empty virus control is plotted for a representative example of assays performed in duplicate.

We also attempted to rescue CP-751,871-induced growth inhibition by enforced expression of a constitutively active IL7Rα mutant (p.L242_L243 insLSRC) which was originally derived from the DND41 cell line[51] and activates downstream signaling by introduction of a cysteine residue in the juxtamembrane region that mediates receptor homodimerization[49]. The insLSRC mutant was unable to rescue growth or to activate AKT in HPB-ALL cells (Fig 8). Taken together, these findings support the conclusion that IGF signaling fulfills an important role in T-ALL cell growth that is distinct from that provided by IL-7, possibly related to a greater perdurance of AKT activation that even a constitutively active IL-7Rα mutant cannot confer.

## Discussion

We and others have observed that while a subset of cell lines indeed show dependence on IGF1R signaling for growth *in vitro*, many do not[25, 31]. Moreover, both IGF1R blocking antibodies and small molecule tyrosine kinase inhibitors have met with limited success in clinical trials such that these are generally considered inactive as single agents[32, 33]. Accordingly, it is useful to consider prospectively what mechanisms might arise by which cells circumvent or compensate for reduced IGF signaling[16, 22]. Prior studies have examined this issue in the context of solid tumors and interestingly different pathways were identified in different tumor types[23, 52], highlighting the importance of cellular context. Herein we have taken up this issue in the context of T-ALL and employed a broad screen of 27 established human T-ALL cell lines to capture a spectrum of genetic backgrounds in the disease. We found low levels of IGF1R expression and PTEN-negative status to correlate with resistance to IGF1R inhibition. Interestingly, knock-down or enforced re-expression of PTEN failed to confer or reverse resistance, respectively, suggesting that other signaling inputs may be required in combination with PTEN to modulate IGF dependence. In fact, our observation that PTEN-negative CCRF-CEM cells are not sensitive to combined inhibition of IGF1R and PI3Kγ suggests that other activatory signals at or above the level of p110α/β/δ are operative in this particular context. We also found PI3K/AKT to be more relevant than RAS/RAF/MEK/ERK in communicating IGF signals to downstream growth/survival effectors, and that trophic signals from IGF and IL-7 are not equivalent in their ability to support T-ALL growth despite both having the ability to activate PI3K/AKT. Of note, we previously reported that patient-derived xenograft T-ALL cells carrying homozygous PTEN frameshift mutations and cultured *ex vivo* in defined media with supplemental IL-7 were sensitive to IGF1R inhibition with CP-751,871[53], further supporting our conclusions that IGF-1 and IL-7 are non-redundant and that PTEN loss in itself does not confer IGF independence. Despite these findings, it is notable that even the most sensitive of cell lines in our panel showed only partial growth inhibition with either CP-751,871 or BMS-754807, suggesting that simultaneous inhibition of additional pathways may be required to achieve clinically meaningful responses.

PI3K/AKT and RAS/RAF/MEK/ERK pathways interact at several nodes including points of both cross-activation and cross-inhibition[34]. The net outcome of these cross-interactions is likely to be highly dependent on cellular context and, given the genetic heterogeneity present even among tumors of the same pathologic subtype, it will likely also differ for each individual patient’s tumor. Nonetheless, several pre-clinical studies have shown that simultaneous inhibition of these two major signaling pathways is effective in certain solid tumors including melanoma, rhadbomyosarcoma, and carcinomas of the colon, ovary, pancreas, and breast [54–56]. Our observation that KrasG12D is unable to rescue growth of IGF inhibitor-sensitive cell lines suggests however that either substantial functional reprogramming or clonal selection may be required in order to create the appropriate context for RAS/RAF/MEK/ERK signaling to replace the trophic effects of PI3K/AKT signaling. Conversely, conditioning or selection under RAS/RAF/MEK/ERK inhibition may be required to drive cells toward PI3K/AKT dependence. Dual inhibitor therapy may thus be important in yielding initial responses in cases where indeed both pathways are actively contributing to cell growth/survival, but in other cases it may be acting more in a pre-emptive fashion to eliminate emerging clones.

Our observation that RAS(G12D) exhibited little if any potency in restoring growth-related phenotypes after IGF1R inhibition begs the question as to what phenotypic advantage is conferred by activated Ras signaling in this context. Indeed, KRAS or NRAS mutations occur frequently in human T-ALL[13, 57–60], while Kras and Notch1 cooperatively induce T-ALL in mouse models[28, 61]. Kras(G12D) perturbs normal thymopoiesis at the DN stage in mice[61, 62], suggesting that activated Ras expands the pool of T-cell progenitors that are susceptible to transformation. As well, it is notable that mouse T-ALL with activated Kras(G12D) show greater sensitivity to MEK inhibition as compared to Kras(WT) tumors, suggesting that activated Ras induces so-called “oncogene addiction”, but is otherwise not generally advantageous in T-ALL.

IL-7 signaling is undoubtedly important for sustaining T-ALL cell growth/survival as highlighted by its requirement for *ex vivo* expansion of primary human T-ALL cells[46, 47, 53]. We found that although IL-7 was able to acutely stimulate AKT to a comparable level as IGF-1, it was unable to maintain this activation at a sufficient level/duration needed to sustain growth under conditions where IGF1R was inhibited. This may suggest negative feedback mechanisms occur downstream of IL7R, but which do not exist for IGF signaling in this context. As well, the occurrence of activating mutations in STAT5B in T-ALL[63] suggests that constitutive activation occurring below the level of JAK/SOCS interaction[64] may be needed to achieve the intensity/duration of downstream signaling required to support tumor propagation.

Expression of MYB has been associated with sensitivity to IGF1R inhibition by CP-751,871 in lung, breast, and colorectal cancer cell lines[65]. In T-ALL, MYB can be overexpressed by translocation or duplication[66, 67] and indeed five cell lines in our panel are known to carry extra chromosomal copies of MYB (ALLSIL, RPMI 8402,MOLT4, P12 Ichikawa, CCRF-CEM)[67]; however, these were not uniformly sensitive to CP-751,871 in our hands suggesting that multiple genetic variables likely contribute to the net pharmacological effect. In summary, our work would support that IGF dependence in T-ALL is characterized by sustained activation of AKT that cannot be not achieved through either PTEN loss or IL-7R activation alone, and is not readily compensated by RAS/RAF/MEK/ERK signaling. Further studies will be required to determine the extent to which these findings apply in primary patient material.

## Supporting Information

S1 Fig Dose titration of CP-751,871 on selected T-ALL cell lines. Cell growth as measured by resazurin reduction assay. Cell lines were cultured *in vitro* for 3 days with the indicated final concentrations of IGF1R blocking antibody (CP-751,871). Mean resorufin (reduced resazurin) fluorescence values +/- SD after normalization to mock-treated controls are plotted for assays performed in triplicate.
(TIF)

S2 Fig BMS-754807 inhibits cell growth to a greater extent than CP-751,871 in a subset of human T-ALL cell lines. Cell growth as measured by resazurin reduction assay. BMS data points significantly different than their corresponding CP data points are indicated in red (p<0.05, t-test). The horizontal dotted line demarcates cell lines with greater than 10% difference between BMS and CP values. *Plotted data are identical to those presented in Fig 1, but normalized to the CP-751,871 fluorescence values*.
(TIF)

S3 Fig Surface IGF1R expression level in human T-ALL cell lines. Surface expression level was measured by flow cytometry following staining of live cells with primary antibody against IGF1R (αIR3) followed by an APC-conjugated secondary antibody. All expression values are normalized to the level exhibited by the HPBALL cell line. Mean fluorescence intensity (MFI) with standard deviation (SD) of the population is plotted in (**A**), and provided in tabular form in (**B**).
(TIF)

S4 Fig Correlation between IGF1R inhibitor sensitivity and IGF1R expression level is lost when cell lines with highest IGF1R expression levels are excluded. Data are identical to that presented in Fig 2, excluding the top 3 IGF1R-expressing cell lines ALL-SIL, HPB-ALL, and SUP-T1.
(TIF)

S5 Fig T-ALL cell lines activate PI3K/AKT signaling in response to IGF1. Flow cytometric analysis for intracellular phospho-AKT levels. PTEN wild-type (ALL-SIL and TALL-1) and PTEN null/mutated (P12 Ichikawa and PF-382) cell lines were serum starved for 20–24 hours, then stimulated with 100 ng/ml IGF1 for 15 minutes. Cells were fixed immediately, permeabilized, stained with anti-phospho-AKT (Ser473) antibody, and analyzed by flow cytometry. Mean fluorescence intensity values were normalized to unstarved cells (“steady state”).
(TIF)

S6 Fig Western blot analysis of exogenously expressed proteins. Whole cell lysates were prepared from HPBALL and TALL-1 cell lines virally transduced with CD8-IGF1R, myrAKT, RAS(G12D), or empty vector (EV) as indicated, separated by SDS-PAGE, and transferred to membranes. The upper blots in panels (**A**), (**B**), and (**C**) were probed with antibodies against IGF1Rβ, AKT, and RAS(G12D), respectively. The corresponding lower blots in each panel were probed with β-actin as a loading control. CCRF-CEM, 144CLP, and P12-Ichikawa cell line samples in panel (**C**) serve as positive staining controls for RAS(G12D). The numbers below the upper panel in (**C**) indicate RAS(G12D) expression level relative to the respective EV controls. *N*.*B*. *TALL-1*, *CCRF-CEM*, *and P12-Ichikawa carry endogenous G12D mutations in NRAS*, *KRAS*, *and NRAS*, *respectively*. *The murine T-ALL cell line*, *144CLP*, *was generated from a Kras(G12D) knock-in mouse*.
(TIF)

S7 Fig Rescue from CP-751,871-induced growth inhibition is dependent on active IGF1R signaling. Cell growth as measured by resazurin reduction. T-ALL cells were transduced with lentiviral vectors as indicated, FACS sorted, and then cultured *in vitro* with IGF1R blocking antibody (1 μg/ml CP-751,871) for 3 days. Mean resorufin fluorescence values +/- SD after normalization to respective mock-treated controls are plotted for assays performed in triplicate. ****, *p<0*.*0001; ns*, *not significant (2-way ANOVA with Sidak’s multiple comparisons test)*.
(TIF)

S8 Fig Lentiviral expression of constitutively active RAS(G12D) in T-ALL cell lines does not activate ERK downstream. (**A,C**) Western blot analysis for ERK phosphorylation. (**A**) T-ALL cell lines were transduced with constitutively active RAS(G12D) lentivirus, FACS sorted, and cultured under standard conditions. (**C**) HPB-ALL cells were serum starved for 24 hours, then pulsed with IGF-1 (100 ng/ml), IL-7 (100 ng/ml), SDF-1 (100 ng/ml), or PMA (100 ng/ml) for 10 minutes, and fixed immediately thereafter with paraformaldehyde. Whole cell lysates were prepared and analyzed by Western blot using anti-phospho-ERK1/2 (T202/Y204) and anti-total ERK2 antibodies. (**B**) Flow cytometric analysis for intracellular phospho-ERK levels. T-ALL cell lines were transduced with constitutively active RAS(G12D) lentivirus with NGFR marker, fixed/permeabilized, and stained with antibodies against phospho-ERK1/2 (T202/Y204) and NGFR. Data are shown for gated live transduced (NGFR+) and untransduced (NGFR-) cells from the same culture.
(TIF)

S9 Fig The constitutively active RAS(G12D) mutant is competent in activating ERK. (**A**) Western blot analysis for ERK phosphorylation. 293T cells were transiently transfected with the RAS(G12D) lentiviral expression construct. Equivalent transfection compared to empty vector control was confirmed by flow cytometry for the linked NGFR marker. Positive control HPB-ALL cells were treated with 100 ng/ml PMA for 10 minutes. Whole cell lysates were analyzed by Western blot using anti-phospho-ERK1/2 (T202/Y204) and anti-total ERK2 antibodies. (**B**) Flow cytometric analysis for intracellular phospho-ERK levels. 293T cells were transiently transfected with the RAS(G12D) lentiviral expression construct, fixed/permeabilized, and stained with antibodies against phospho-ERK1/2 (T202/Y204) and NGFR. Data are shown for gated live transduced (NGFR+) and untransduced (NGFR-) cells from the same culture.
(TIF)

S10 Fig Steady-state AKT activation level correlates with cell size. Flow cytometric analysis for intracellular phospho-AKT levels. (**A,C**) Cells were cultured *in vitro* with IGF1R blocking antibody (1 μg/ml CP-751,871) for 3 days. (**B,D**) Cells were transduced with lentiviral constructs as indicated. Cells were harvested, fixed/permeabilized, and stained with antibodies against phospho-AKT (Ser473) in (**A,C**) and also against NGFR in (**B,D**). Mean fluorescence intensity values are plotted after normalization to mock-treated cells in (**A,C**), or to untransduced cells within each of the cultures, then scaling to the empty virus control in (**B,D**). Representative examples of assays performed in duplicate are depicted.
(TIF)

S11 Fig T-ALL cell lines show activation of PI3K/AKT, but not MAPK/ERK following stimulation with IGF1. The indicated human T-ALL cell lines were serum starved overnight, then pulsed for 10 minutes with recombinant human IGF1. Cells were fixed immediately thereafter, then permeabilized and stained with AF647-conjugated antibodies against phospho-AKT (pAKT) or phospho-ERK (pERK), or isotype control. Positive staining controls for pAKT and pERK were HPBALL cells transduced with myrAKT or stimulated with 100 ng/ml PMA, respectively.
(TIF)

S12 Fig PTEN protein status in human T-ALL cell lines. Western blot analysis for PTEN in cell lines whose PTEN status was not previously reported. HPB-ALL is included as a positive staining control. β-actin is shown as a loading control.
(TIF)

S13 Fig Surface IGF1R expression level does not differ significantly between PTEN-positive and PTEN-negative cell lines. Plot of surface IGF1R expression level (mean fluorescence intensity as measured by flow cytometry from S3 Fig) among the 26 cell lines for which PTEN status was available (see S2 Table). Data are identical to that depicted in Fig 2, but here divided into PTEN-positive and PTEN-negative subsets. *ns*, *not significant (t test)*.
(TIF)

S14 Fig Correlation between IGF1R inhibitor sensitivity and IGF1R expression level is lost when cell lines with highest IGF1R expression levels are excluded. Data are identical to that presented in Fig 4, excluding the top 3 IGF1R-expressing cell lines ALL-SIL, HPB-ALL, and SUP-T1.
(TIF)

S15 Fig Lentiviral expression and knock-down of PTEN in T-ALL cell lines. Western blot analysis for PTEN protein expression level. Cell lines were transduced with the indicated lentiviral constructs and FACS sorted prior to preparation of whole cell lysates. ZAP-70 is shown as a loading control.
(TIF)

S16 Fig Exogenous, lentivirally expressed PTEN is functionally active in PTEN-negative cell lines P12 Ichikawa and PF-382. Flow cytometric analysis for intracellular phospho-AKT levels. Cells were transduced with PTEN lentivirus, then harvested and stained with anti-phospho-AKT (Ser473) antibody. Data are shown for gated live transduced (GFP+) and untransduced (GFP-) cells from the same culture. Plotted values are mean fluorescence intensity after normalization to respective untransduced cell controls.
(TIF)

S17 Fig IL-7 does not rescue HPB-ALL cells from growth inhibition by IGF1R blocking antibody. Cell growth as measured by resazurin reduction assay. HPB-ALL cells were cultured *in vitro* with IGF1R blocking antibody (1 μg/ml CP-751,871) with or without supplemental recombinant IL-7 (100 ng/ml) added daily for 3 days. Mean resorufin fluorescence values +/- SD after normalization to untreated control are plotted for assays performed in triplicate. *ns*, *not significant (2-way ANOVA with Sidak’s multiple comparisons test*).
(TIF)

S18 Fig IL-7 activates STAT5, but not ERK signaling in HPB-ALL cells. Flow cytometric analysis for intracellular phospho-STAT5 and phospho-ERK levels. HPB-ALL cells were serum starved for 24 hours, pulsed with 100 ng/ml recombinant IL-7 (Peprotech), then fixed/permeabilized, and stained with (**A**) anti-phospho-STAT5 (Y694) or (**B**) anti-phosphoERK1/2 (T202/Y204) antibodies. In (**A**), cells were also treated in the last hour of serum starvation (immediately prior to IL-7 pulse) with IGF1R blocking antibody (1 μg/ml CP-751,871).
(TIF)

S1 Table Cell line mutations in PI3K/AKT and MAPK pathways as documented in COSMIC^1^ and CCLE^2^ databases. Coding sequence mutations were filtered by the following criteria: Inclusion in the Cancer Gene Census list (http://cancer.sanger.ac.uk/census); predicted to be pathogenic by the FATHMM-MLK algorithm (http://fathmm.biocompute.org.uk/); and involvement in PI3K/AKT and/or MAPK signaling pathways. 1. http://cancer.sanger.ac.uk/cosmic. 2. http://www.broadinstitute.org/ccle.
(DOCX)

S2 Table PTEN protein expression and IL-7Rα mutational status in human T-ALL cell lines. ND, not determined.
(DOCX)
