# Supplementary figures and images for: IGF1R Derived PI3K/AKT Signaling Maintains Growth in a Subset of Human T-Cell Acute Lymphoblastic Leukemias
Source: PLoS One. 2016 Aug 17;11(8):e0161158. doi: 10.1371/journal.pone.0161158 (PMC4988785; doi:10.1371/journal.pone.0161158)

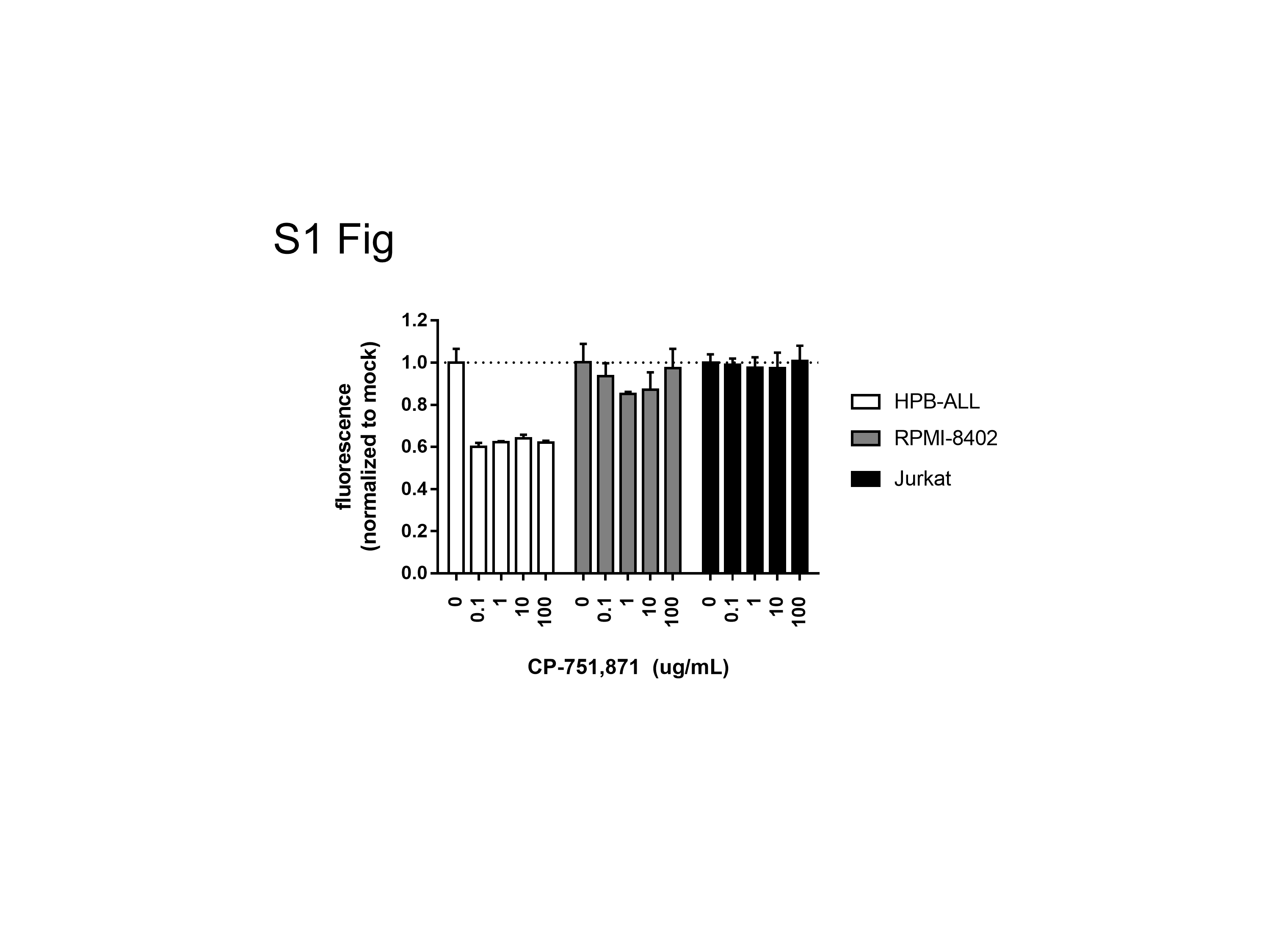

Supplement: S1 Fig — Cell growth as measured by resazurin reduction assay. Cell lines were cultured in vitro for 3 days with the indicated final concentrations of IGF1R blocking antibody (CP-751,871). Mean resorufin (reduced resazurin) fluorescence values +/- SD after normalization to mock-treated controls are plotted for assays performed in triplicate. (TIF) [file pone.0161158.s001.TIF]

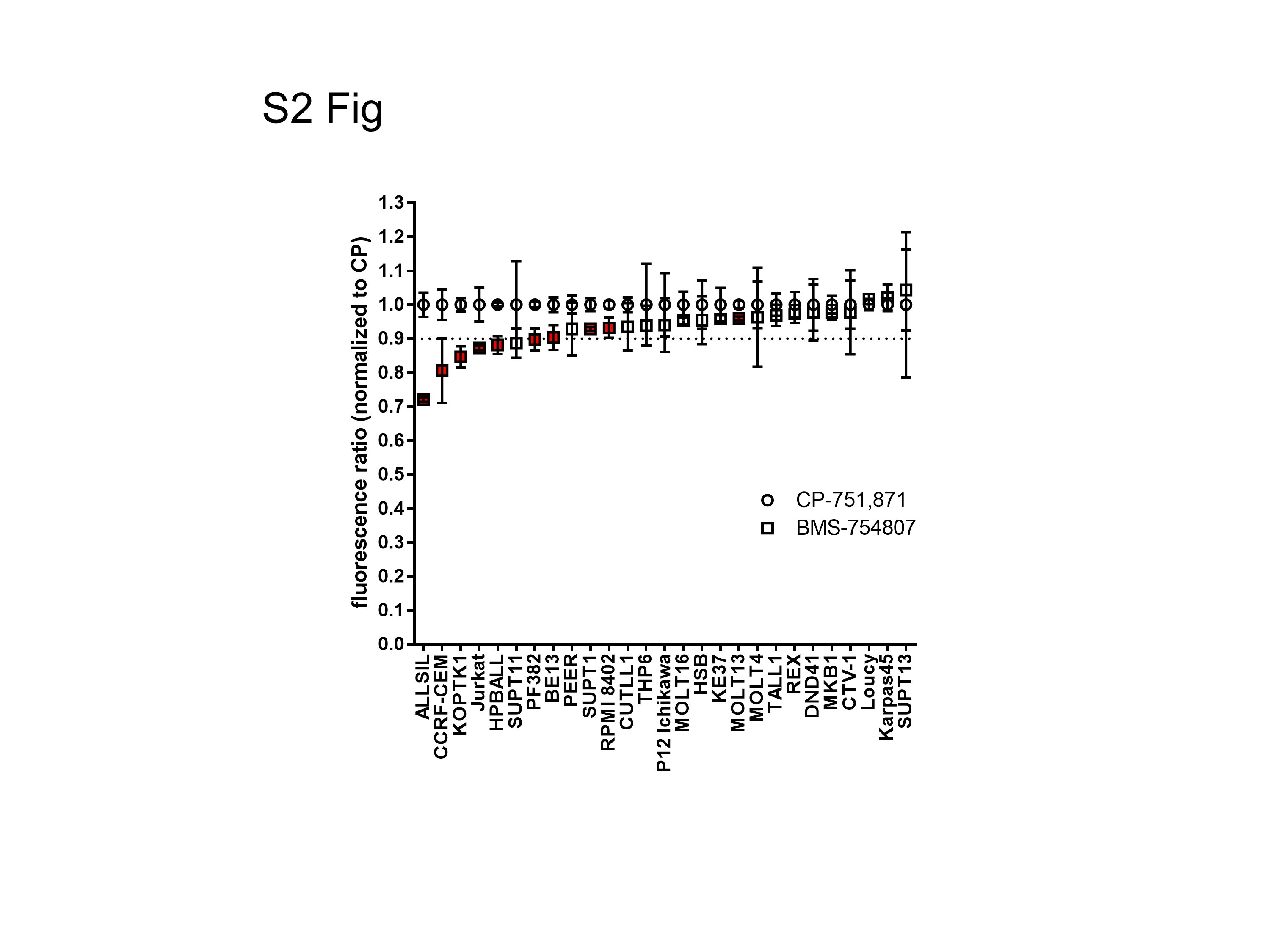

Supplement: S2 Fig — Cell growth as measured by resazurin reduction assay. BMS data points significantly different than their corresponding CP data points are indicated in red (p<0.05, t-test). The horizontal dotted line demarcates cell lines with greater than 10% difference between BMS and CP values. Plotted data are identical to those presented in Fig 1, but normalized to the CP-751,871 fluorescence values. (TIF) [file pone.0161158.s002.TIF]

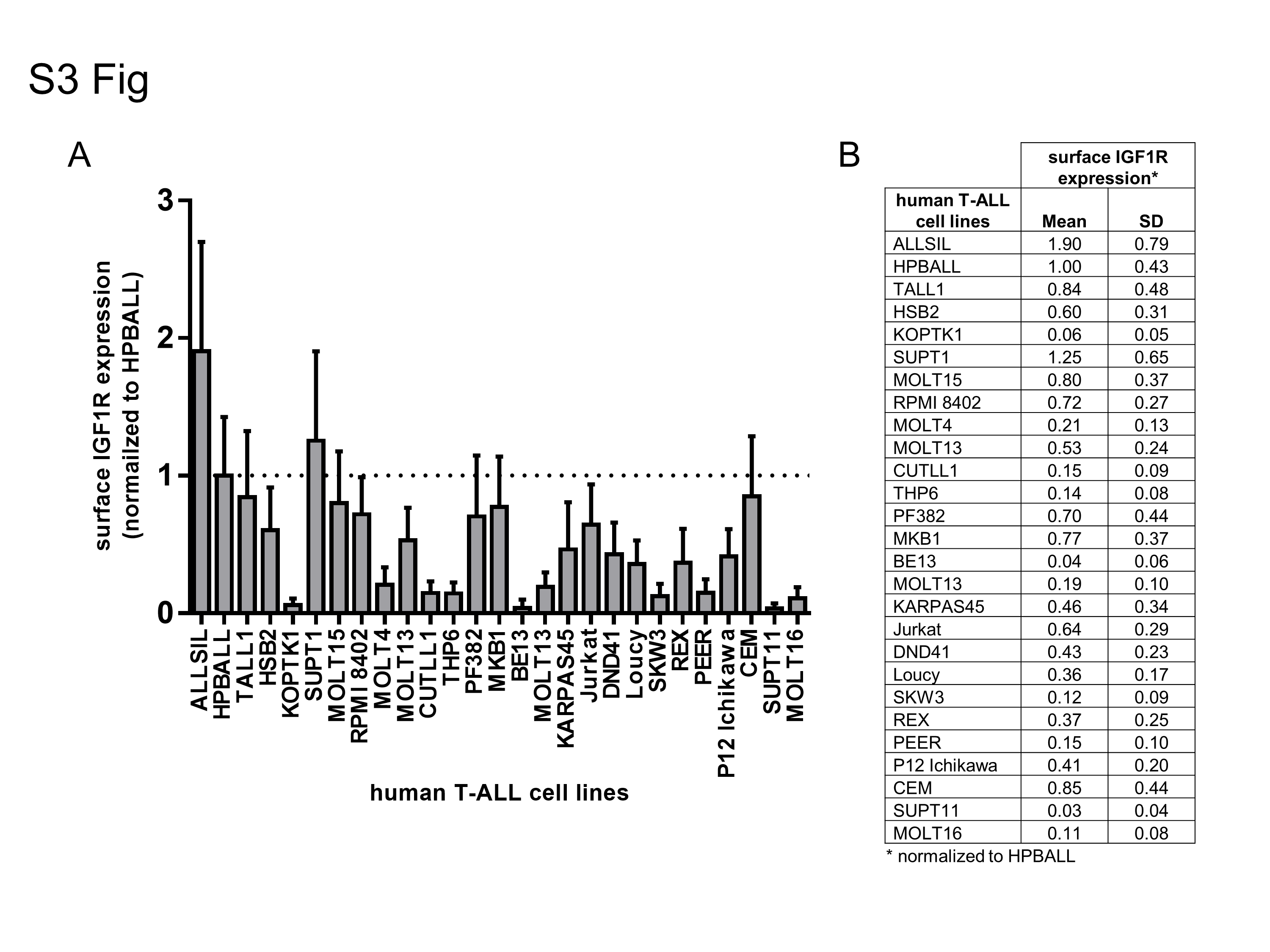

Supplement: S3 Fig — Surface expression level was measured by flow cytometry following staining of live cells with primary antibody against IGF1R (αIR3) followed by an APC-conjugated secondary antibody. All expression values are normalized to the level exhibited by the HPBALL cell line. Mean fluorescence intensity (MFI) with standard deviation (SD) of the population is plotted in (A), and provided in tabular form in (B). (TIF) [file pone.0161158.s003.tif]

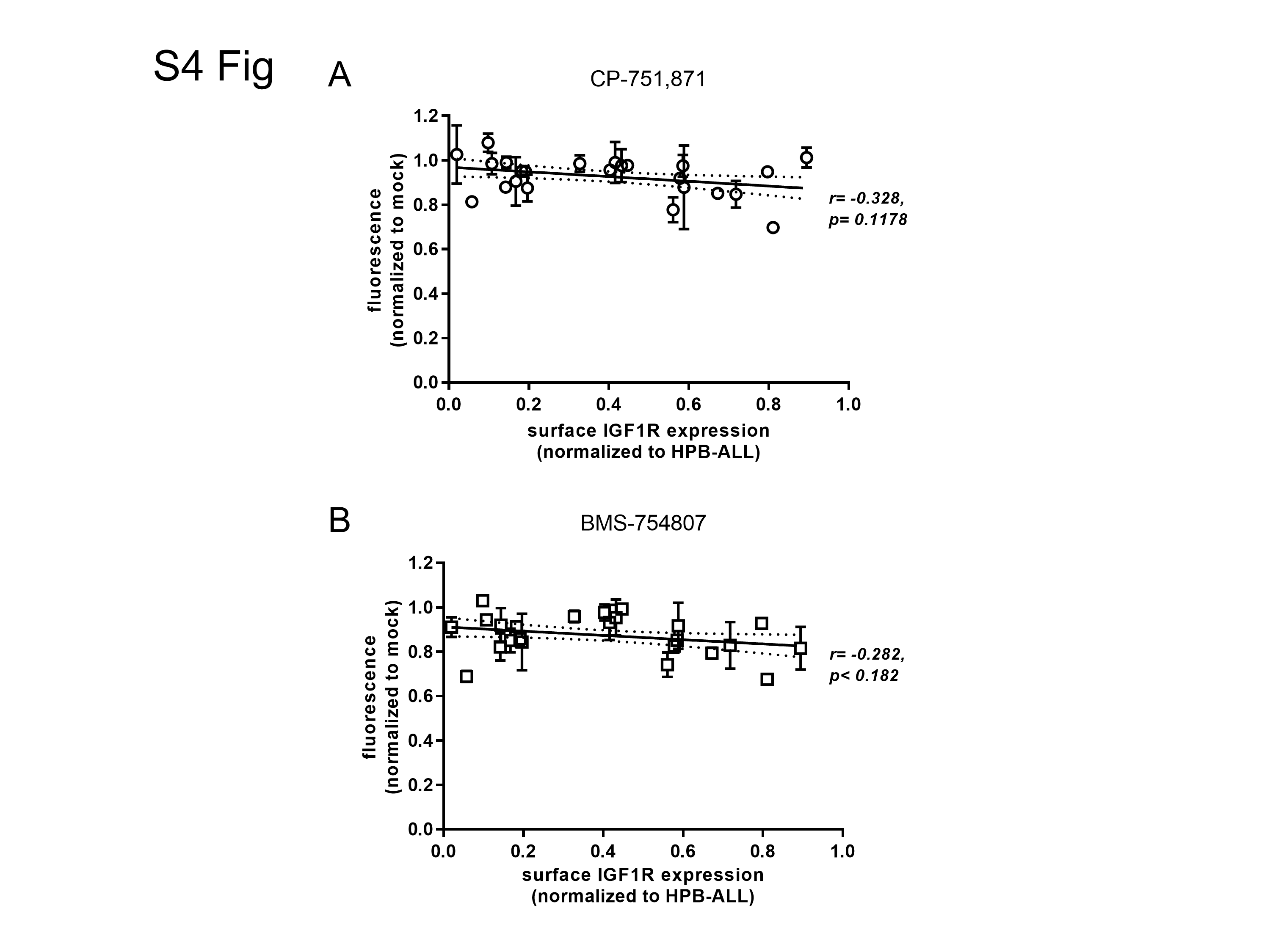

Supplement: S4 Fig — Data are identical to that presented in Fig 2, excluding the top 3 IGF1R-expressing cell lines ALL-SIL, HPB-ALL, and SUP-T1. (TIF) [file pone.0161158.s004.TIF]

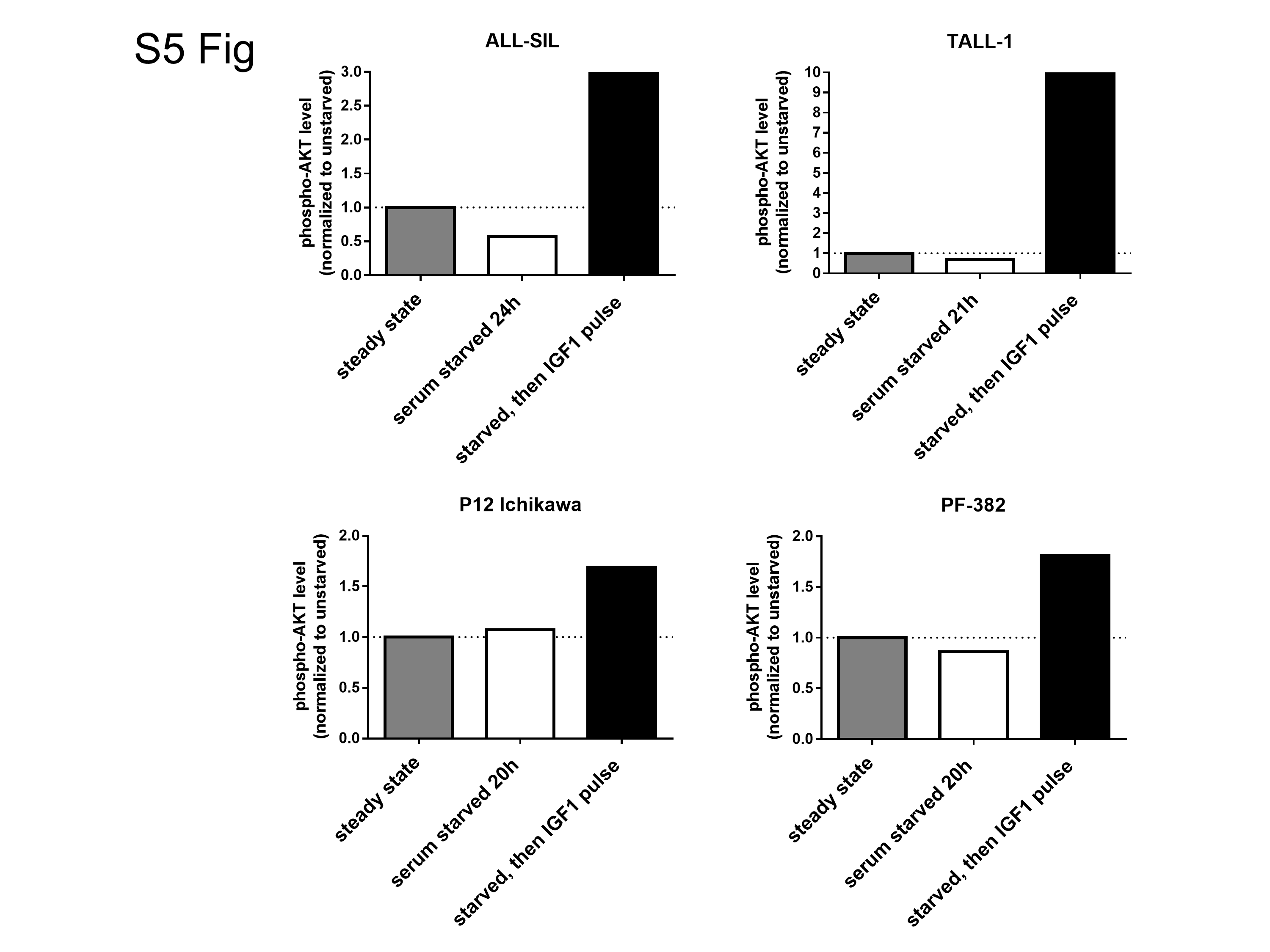

Supplement: S5 Fig — Flow cytometric analysis for intracellular phospho-AKT levels. PTEN wild-type (ALL-SIL and TALL-1) and PTEN null/mutated (P12 Ichikawa and PF-382) cell lines were serum starved for 20–24 hours, then stimulated with 100 ng/ml IGF1 for 15 minutes. Cells were fixed immediately, permeabilized, stained with anti-phospho-AKT (Ser473) antibody, and analyzed by flow cytometry. Mean fluorescence intensity values were normalized to unstarved cells (“steady state”). (TIF) [file pone.0161158.s005.TIF]

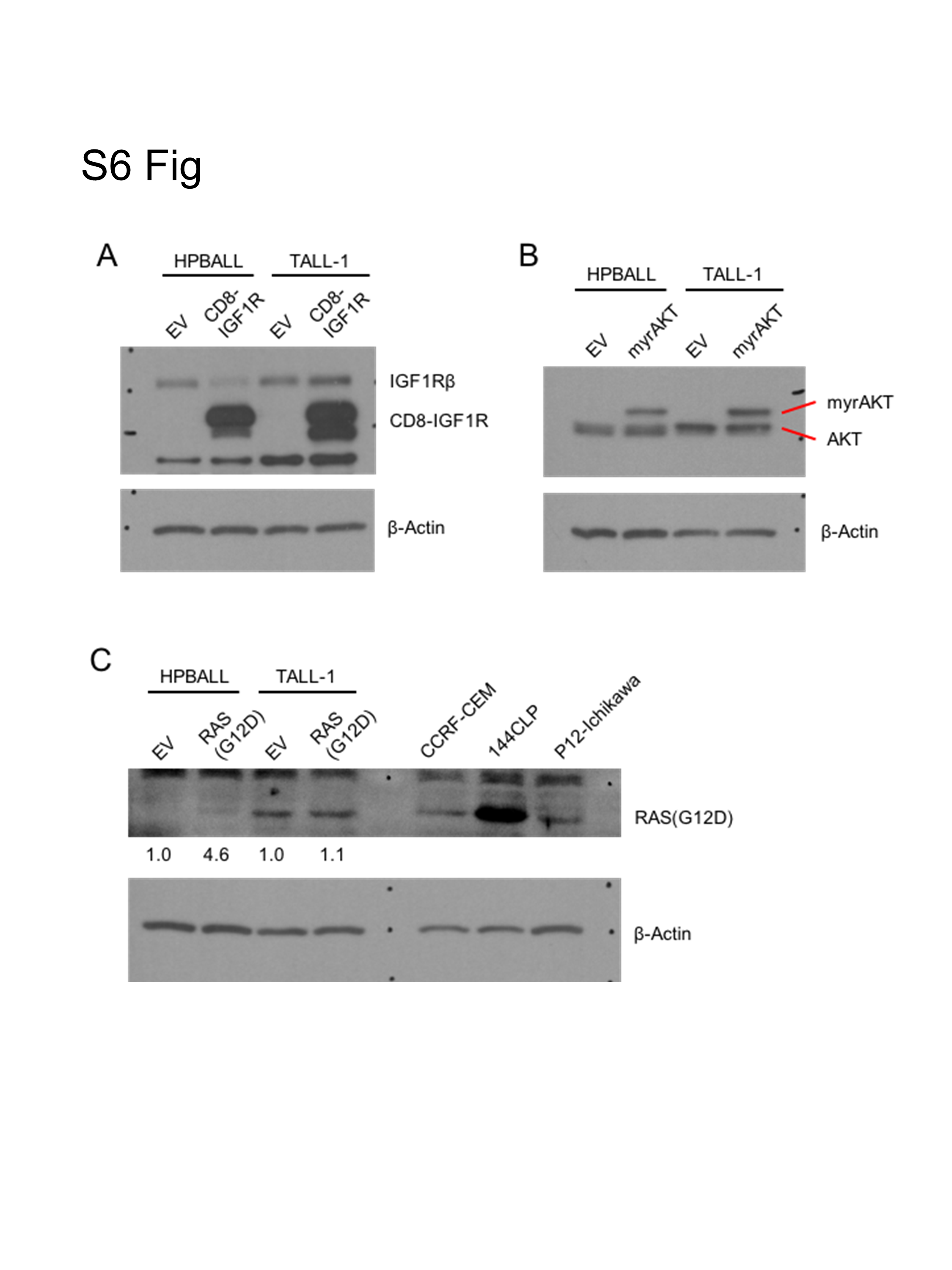

Supplement: S6 Fig — Whole cell lysates were prepared from HPBALL and TALL-1 cell lines virally transduced with CD8-IGF1R, myrAKT, RAS(G12D), or empty vector (EV) as indicated, separated by SDS-PAGE, and transferred to membranes. The upper blots in panels (A), (B), and (C) were probed with antibodies against IGF1Rβ, AKT, and RAS(G12D), respectively. The corresponding lower blots in each panel were probed with β-actin as a loading control. CCRF-CEM, 144CLP, and P12-Ichikawa cell line samples in panel (C) serve as positive staining controls for RAS(G12D). The numbers below the upper panel in (C) indicate RAS(G12D) expression level relative to the respective EV controls. N.B. TALL-1, CCRF-CEM, and P12-Ichikawa carry endogenous G12D mutations in NRAS, KRAS, and NRAS, respectively. The murine T-ALL cell line, 144CLP, was generated from a Kras(G12D) knock-in mouse. (TIF) [file pone.0161158.s006.tif]

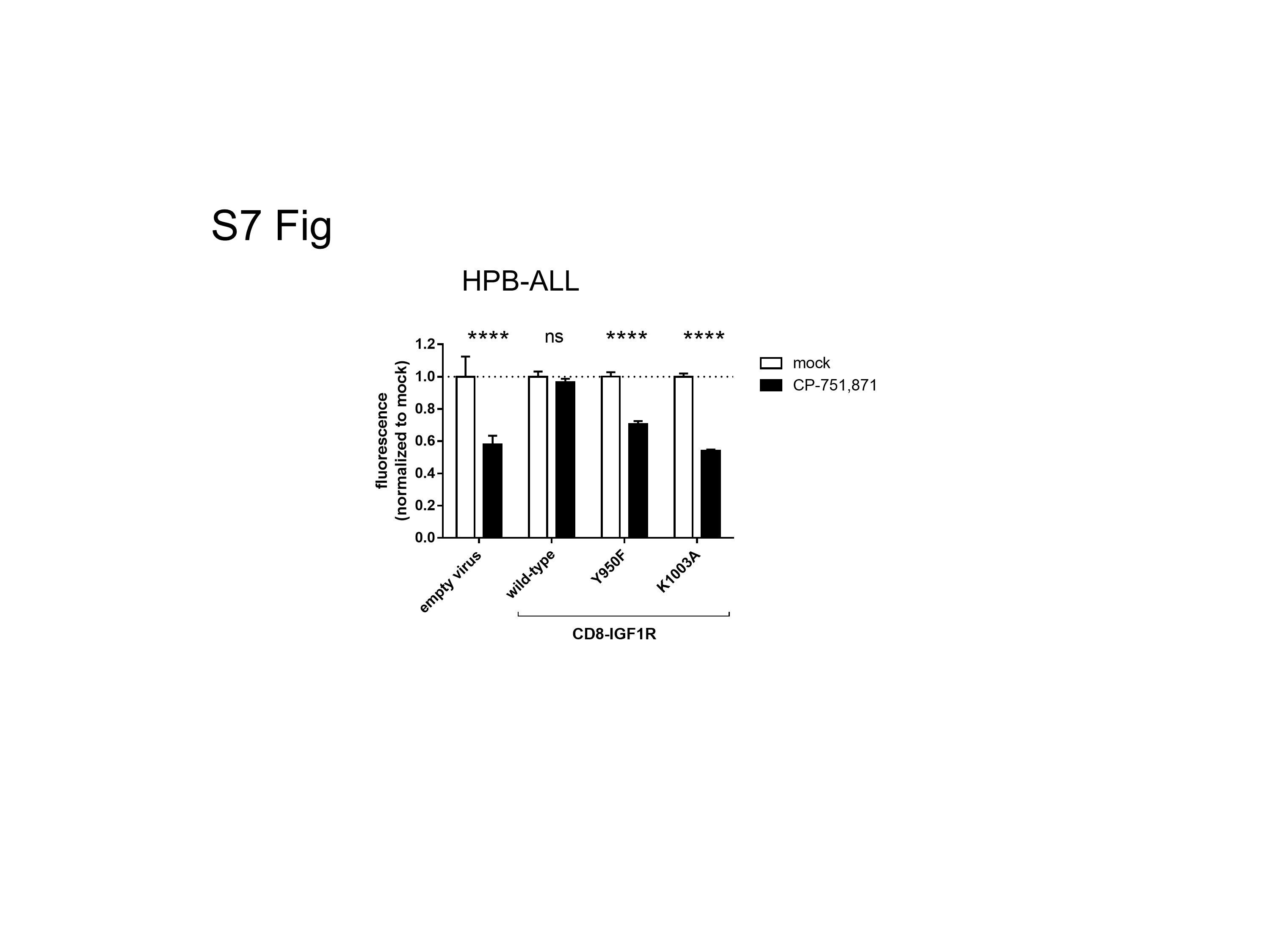

Supplement: S7 Fig — Cell growth as measured by resazurin reduction. T-ALL cells were transduced with lentiviral vectors as indicated, FACS sorted, and then cultured in vitro with IGF1R blocking antibody (1 μg/ml CP-751,871) for 3 days. Mean resorufin fluorescence values +/- SD after normalization to respective mock-treated controls are plotted for assays performed in triplicate. ****, p<0.0001; ns, not significant (2-way ANOVA with Sidak’s multiple comparisons test). (TIF) [file pone.0161158.s007.TIF]

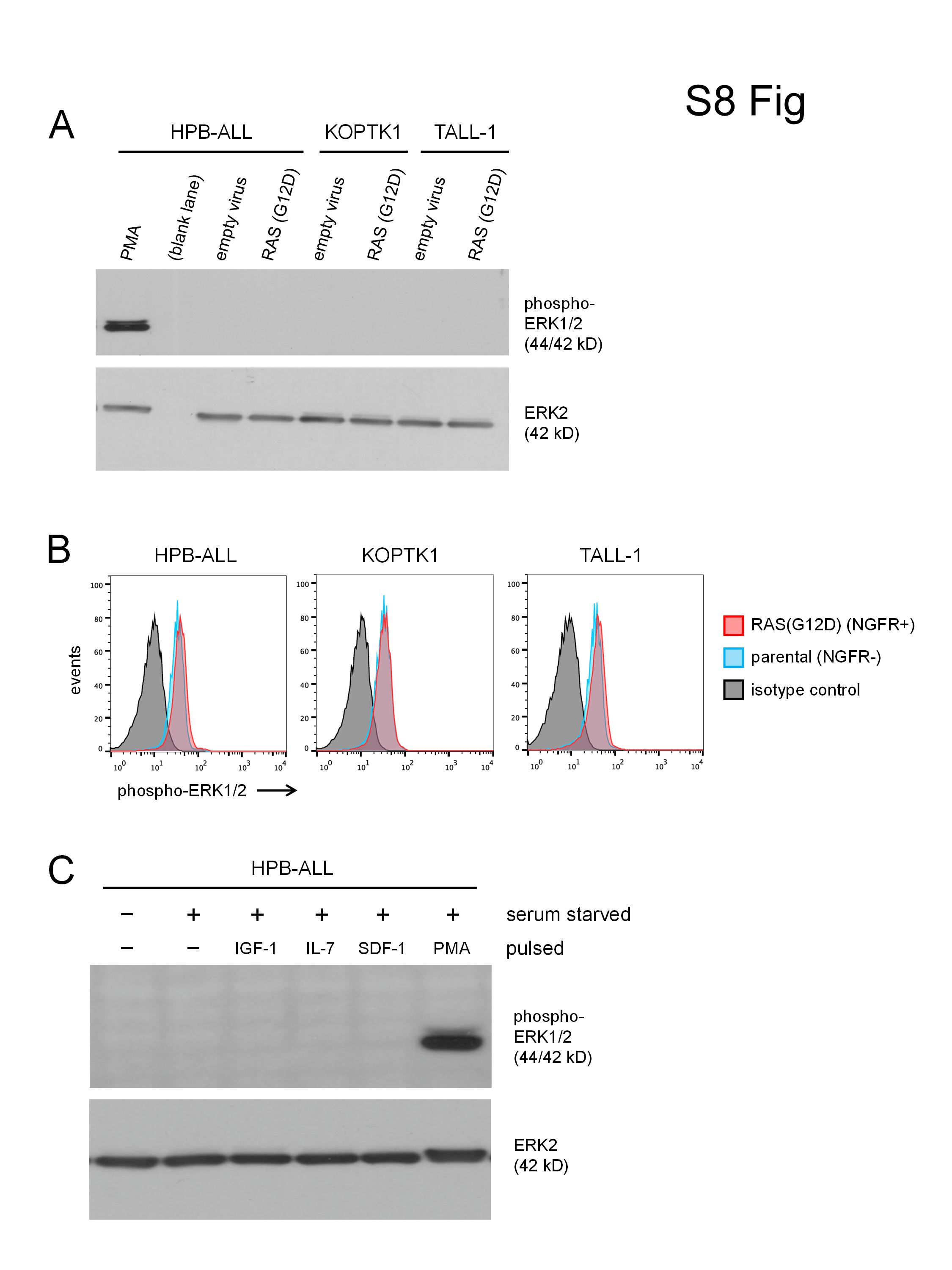

Supplement: S8 Fig — (A,C) Western blot analysis for ERK phosphorylation. (A) T-ALL cell lines were transduced with constitutively active RAS(G12D) lentivirus, FACS sorted, and cultured under standard conditions. (C) HPB-ALL cells were serum starved for 24 hours, then pulsed with IGF-1 (100 ng/ml), IL-7 (100 ng/ml), SDF-1 (100 ng/ml), or PMA (100 ng/ml) for 10 minutes, and fixed immediately thereafter with paraformaldehyde. Whole cell lysates were prepared and analyzed by Western blot using anti-phospho-ERK1/2 (T202/Y204) and anti-total ERK2 antibodies. (B) Flow cytometric analysis for intracellular phospho-ERK levels. T-ALL cell lines were transduced with constitutively active RAS(G12D) lentivirus with NGFR marker, fixed/permeabilized, and stained with antibodies against phospho-ERK1/2 (T202/Y204) and NGFR. Data are shown for gated live transduced (NGFR+) and untransduced (NGFR-) cells from the same culture. (TIF) [file pone.0161158.s008.tif]

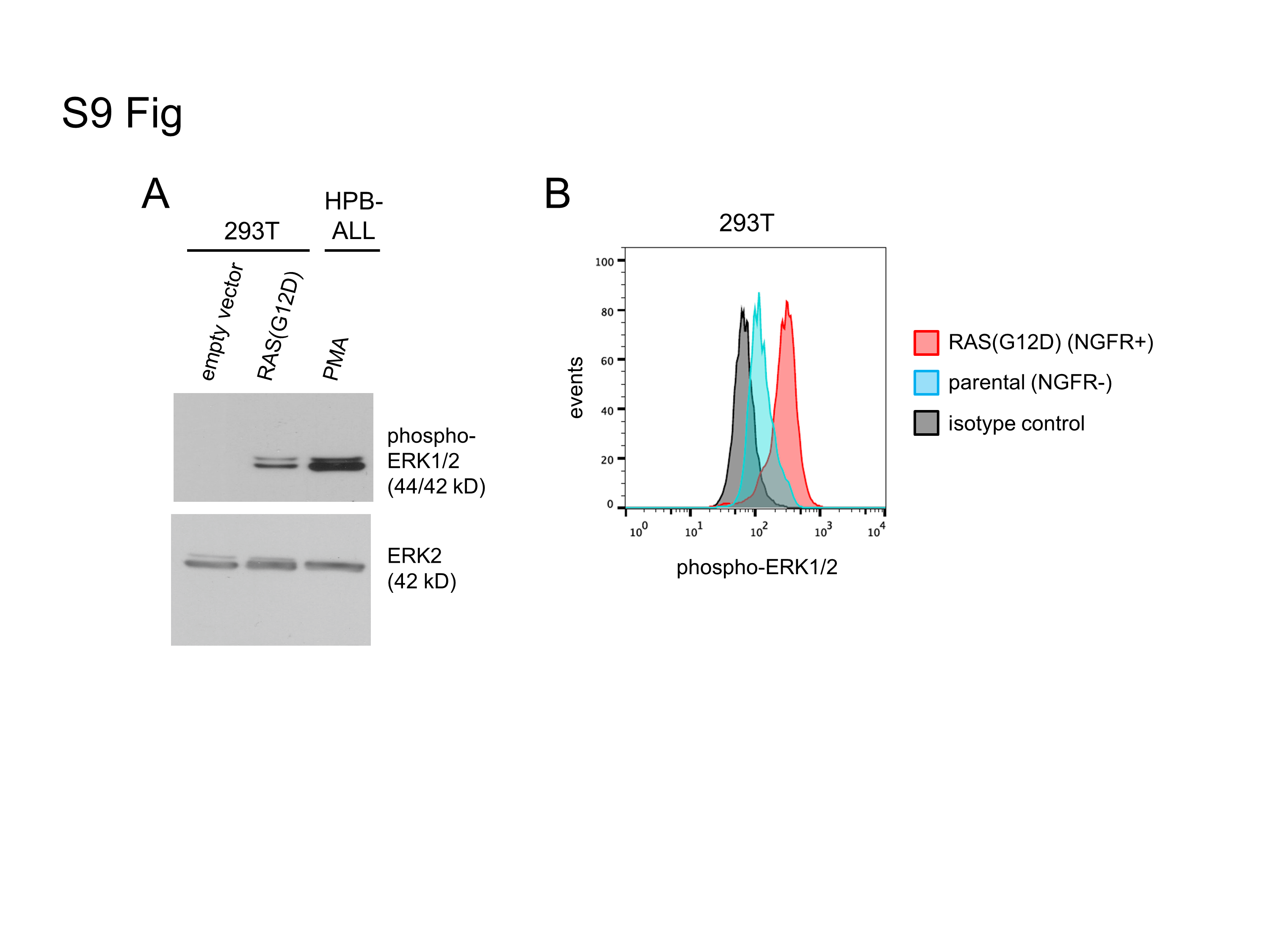

Supplement: S9 Fig — (A) Western blot analysis for ERK phosphorylation. 293T cells were transiently transfected with the RAS(G12D) lentiviral expression construct. Equivalent transfection compared to empty vector control was confirmed by flow cytometry for the linked NGFR marker. Positive control HPB-ALL cells were treated with 100 ng/ml PMA for 10 minutes. Whole cell lysates were analyzed by Western blot using anti-phospho-ERK1/2 (T202/Y204) and anti-total ERK2 antibodies. (B) Flow cytometric analysis for intracellular phospho-ERK levels. 293T cells were transiently transfected with the RAS(G12D) lentiviral expression construct, fixed/permeabilized, and stained with antibodies against phospho-ERK1/2 (T202/Y204) and NGFR. Data are shown for gated live transduced (NGFR+) and untransduced (NGFR-) cells from the same culture. (TIF) [file pone.0161158.s009.TIF]

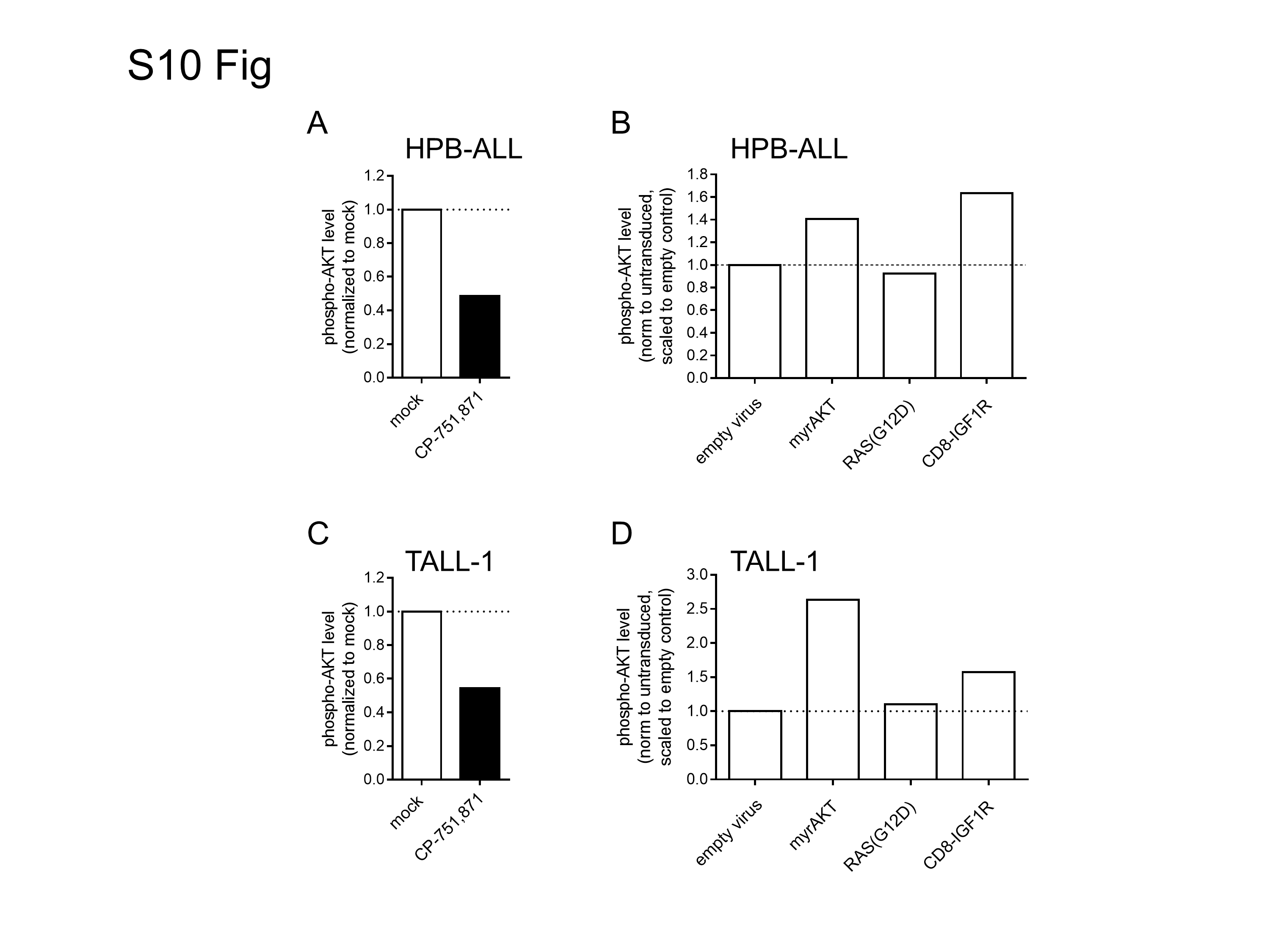

Supplement: S10 Fig — Flow cytometric analysis for intracellular phospho-AKT levels. (A,C) Cells were cultured in vitro with IGF1R blocking antibody (1 μg/ml CP-751,871) for 3 days. (B,D) Cells were transduced with lentiviral constructs as indicated. Cells were harvested, fixed/permeabilized, and stained with antibodies against phospho-AKT (Ser473) in (A,C) and also against NGFR in (B,D). Mean fluorescence intensity values are plotted after normalization to mock-treated cells in (A,C), or to untransduced cells within each of the cultures, then scaling to the empty virus control in (B,D). Representative examples of assays performed in duplicate are depicted. (TIF) [file pone.0161158.s010.TIF]

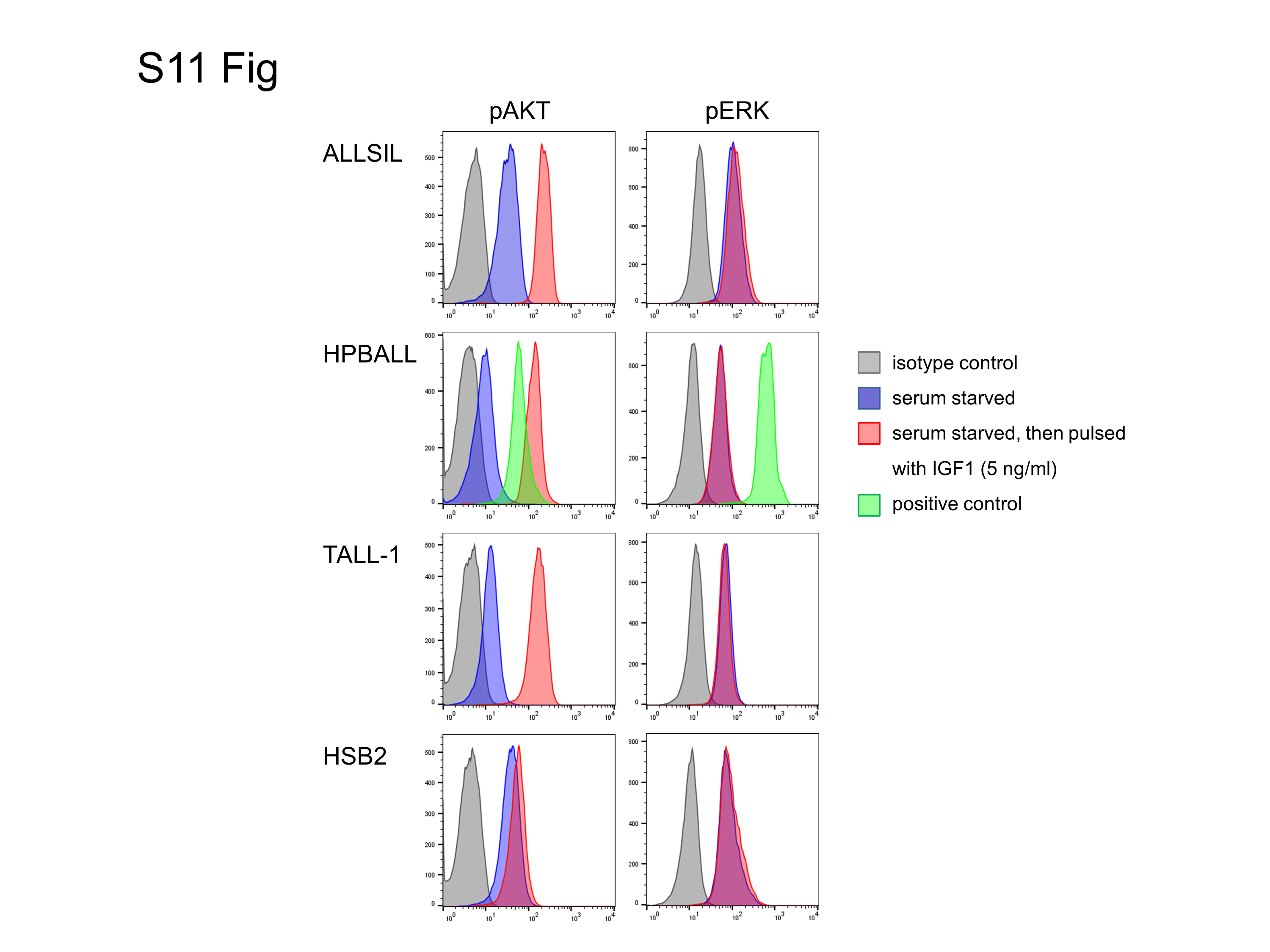

Supplement: S11 Fig — The indicated human T-ALL cell lines were serum starved overnight, then pulsed for 10 minutes with recombinant human IGF1. Cells were fixed immediately thereafter, then permeabilized and stained with AF647-conjugated antibodies against phospho-AKT (pAKT) or phospho-ERK (pERK), or isotype control. Positive staining controls for pAKT and pERK were HPBALL cells transduced with myrAKT or stimulated with 100 ng/ml PMA, respectively. (TIF) [file pone.0161158.s011.tif]

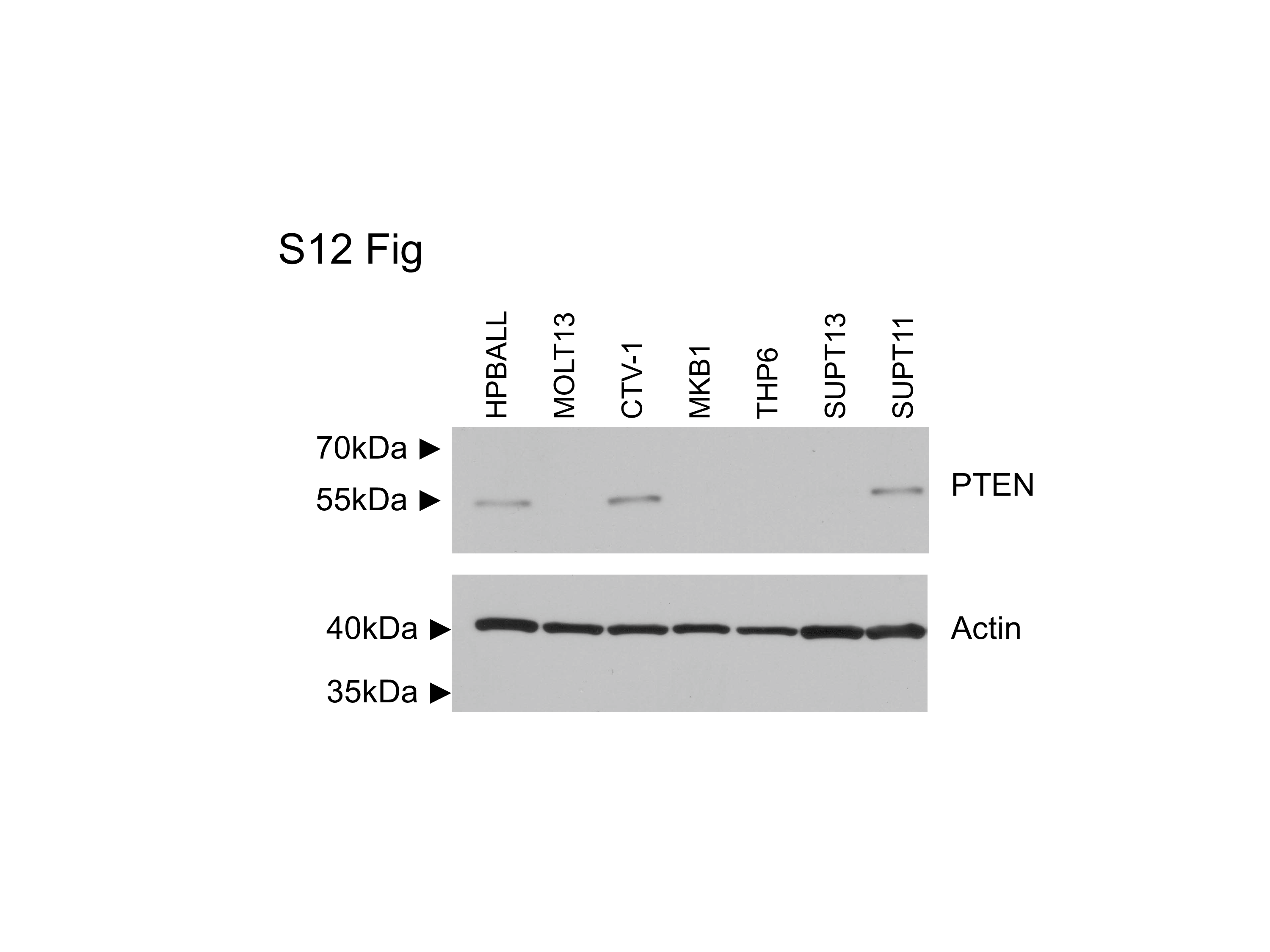

Supplement: S12 Fig — Western blot analysis for PTEN in cell lines whose PTEN status was not previously reported. HPB-ALL is included as a positive staining control. β-actin is shown as a loading control. (TIF) [file pone.0161158.s012.TIF]

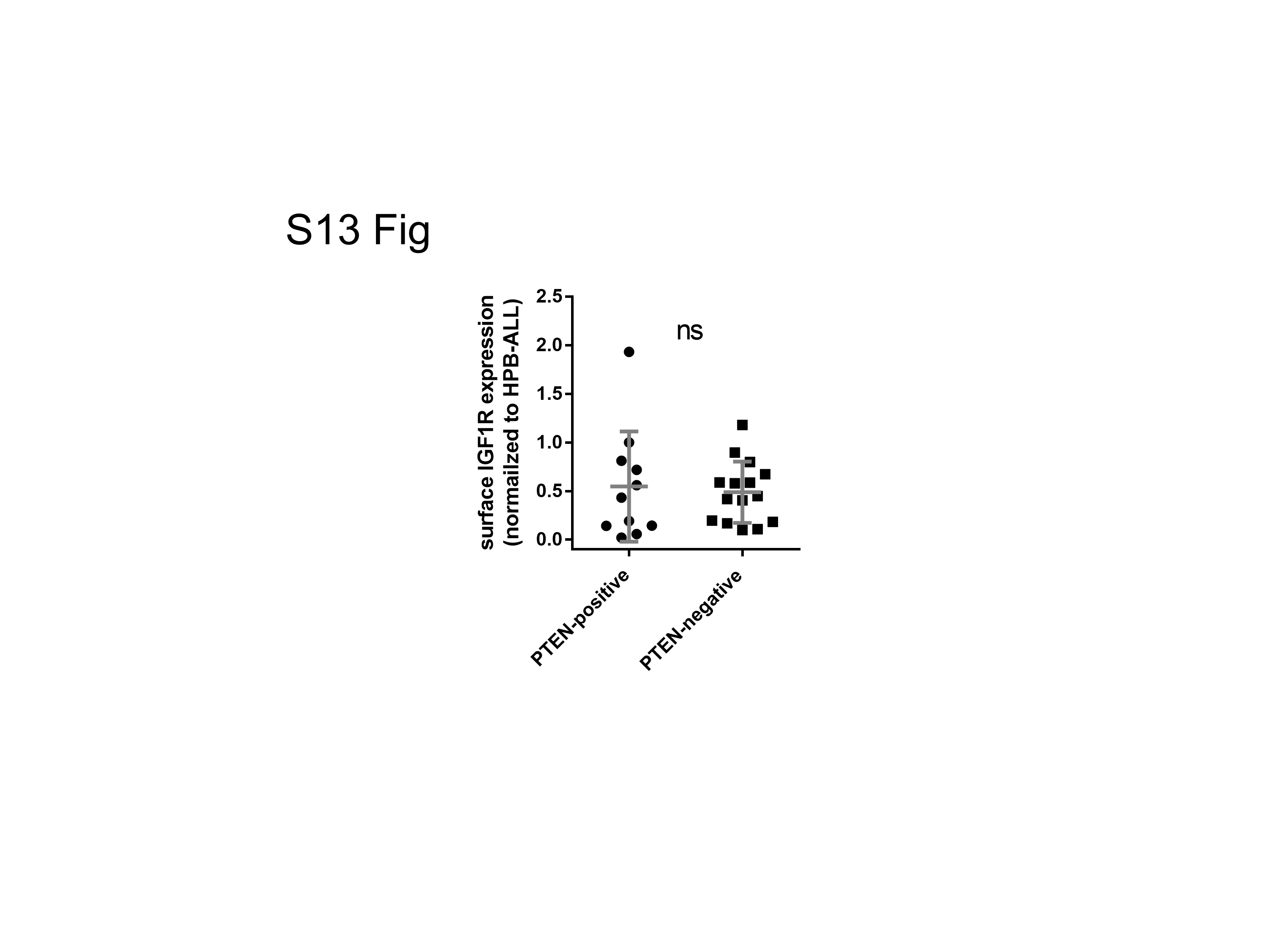

Supplement: S13 Fig — Plot of surface IGF1R expression level (mean fluorescence intensity as measured by flow cytometry from S3 Fig) among the 26 cell lines for which PTEN status was available (see S2 Table). Data are identical to that depicted in Fig 2, but here divided into PTEN-positive and PTEN-negative subsets. ns, not significant (t test). (TIF) [file pone.0161158.s013.TIF]

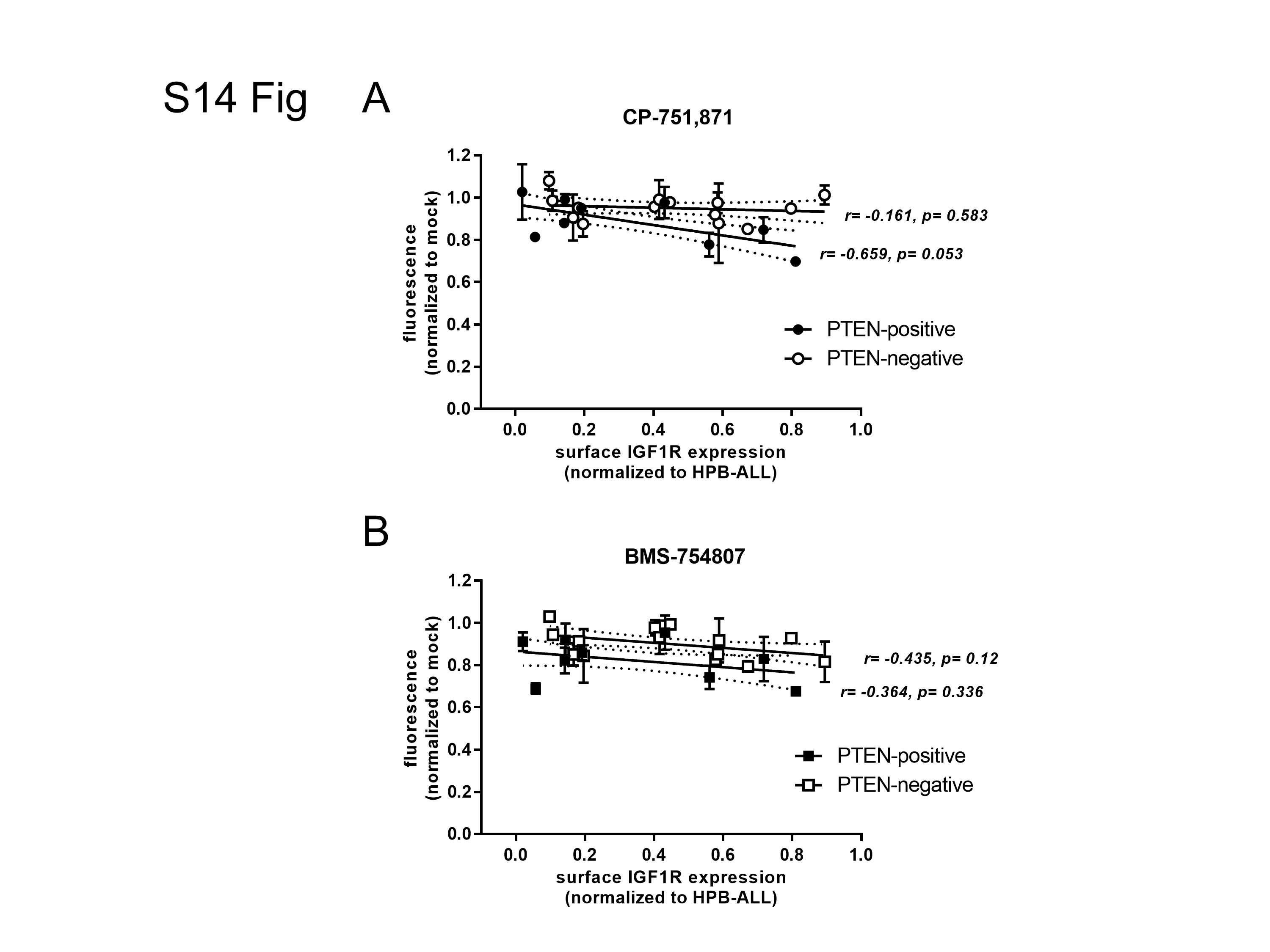

Supplement: S14 Fig — Data are identical to that presented in Fig 4, excluding the top 3 IGF1R-expressing cell lines ALL-SIL, HPB-ALL, and SUP-T1. (TIF) [file pone.0161158.s014.TIF]

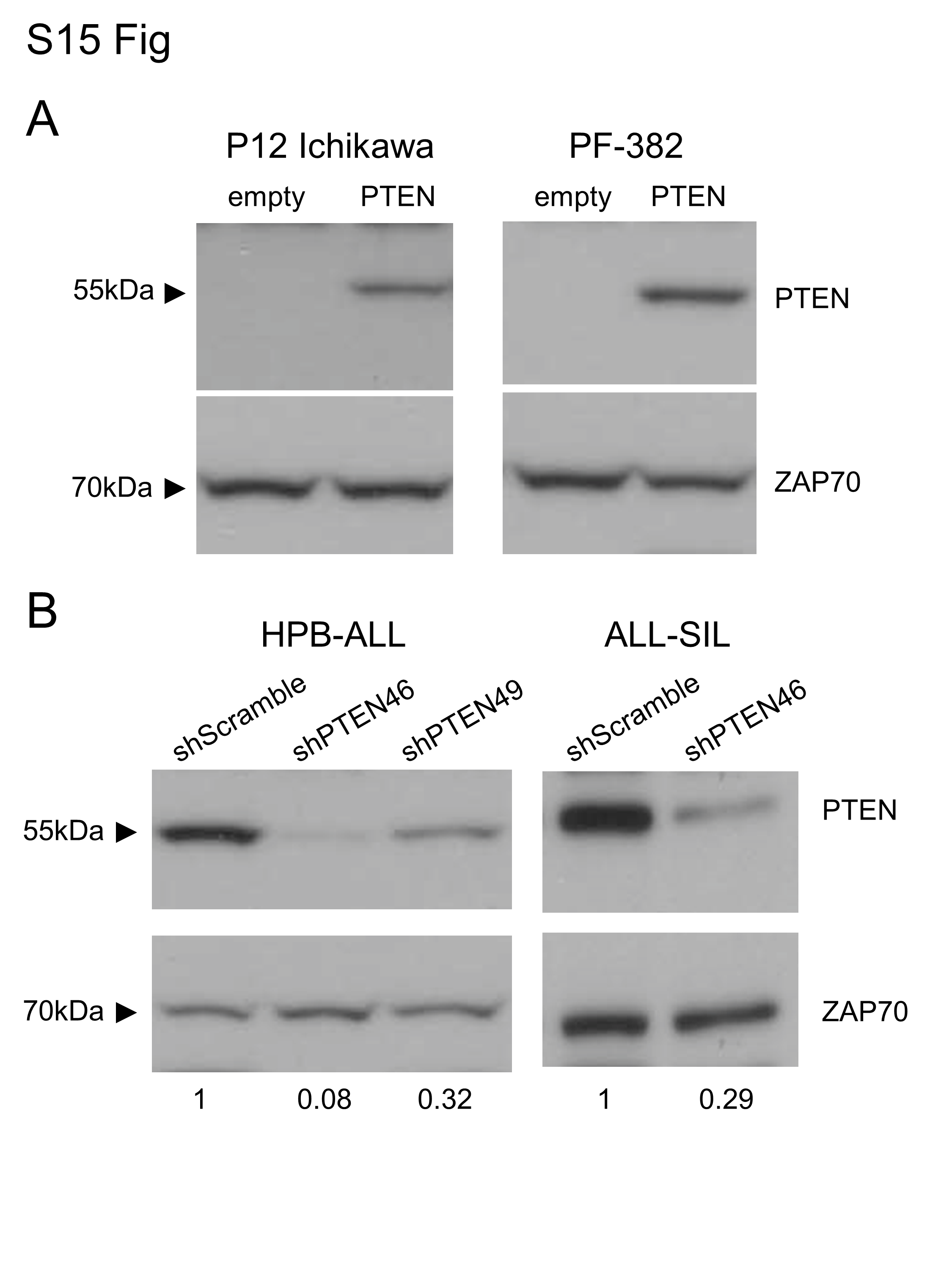

Supplement: S15 Fig — Western blot analysis for PTEN protein expression level. Cell lines were transduced with the indicated lentiviral constructs and FACS sorted prior to preparation of whole cell lysates. ZAP-70 is shown as a loading control. (TIF) [file pone.0161158.s015.tif]

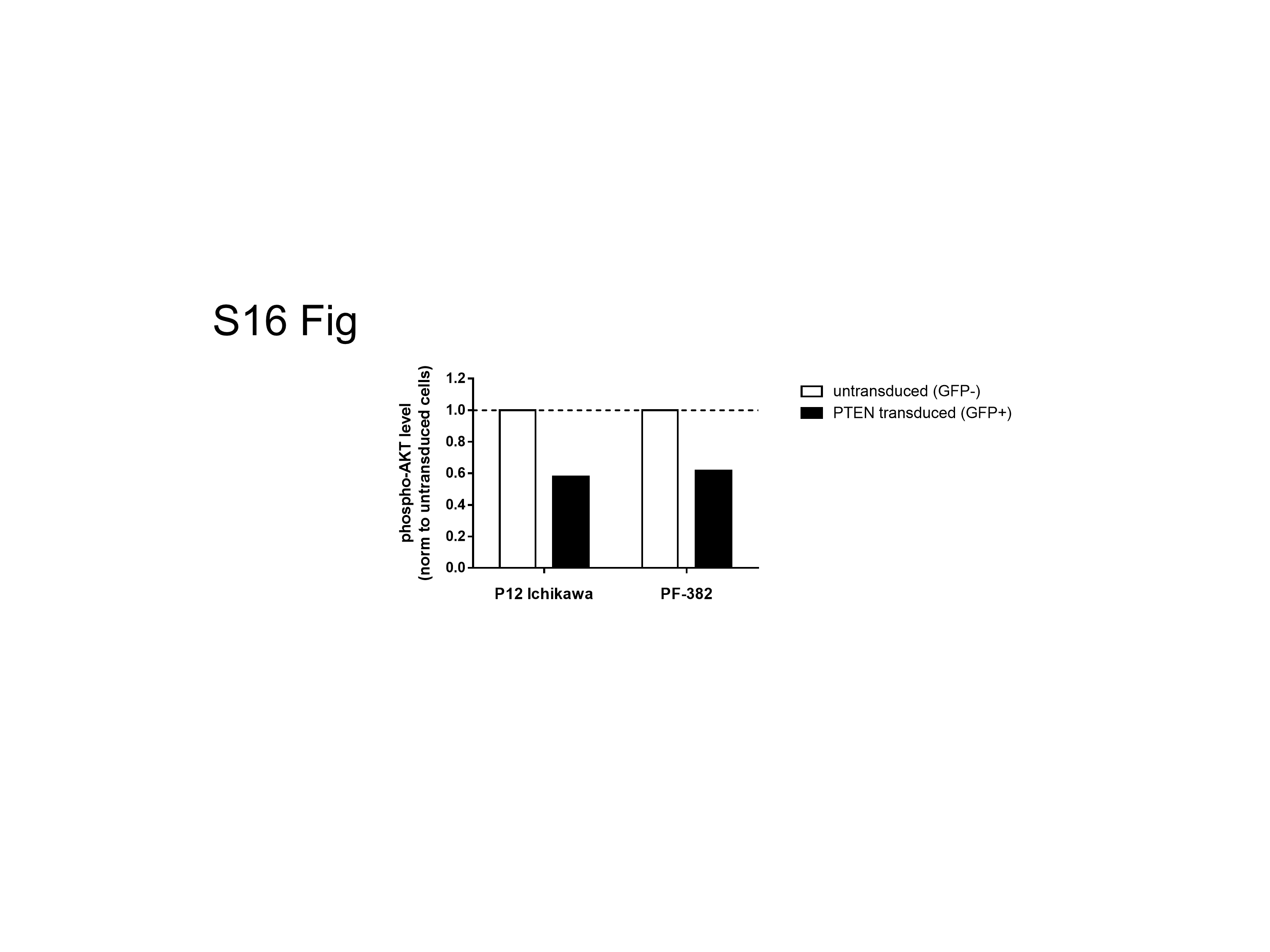

Supplement: S16 Fig — Flow cytometric analysis for intracellular phospho-AKT levels. Cells were transduced with PTEN lentivirus, then harvested and stained with anti-phospho-AKT (Ser473) antibody. Data are shown for gated live transduced (GFP+) and untransduced (GFP-) cells from the same culture. Plotted values are mean fluorescence intensity after normalization to respective untransduced cell controls. (TIF) [file pone.0161158.s016.TIF]

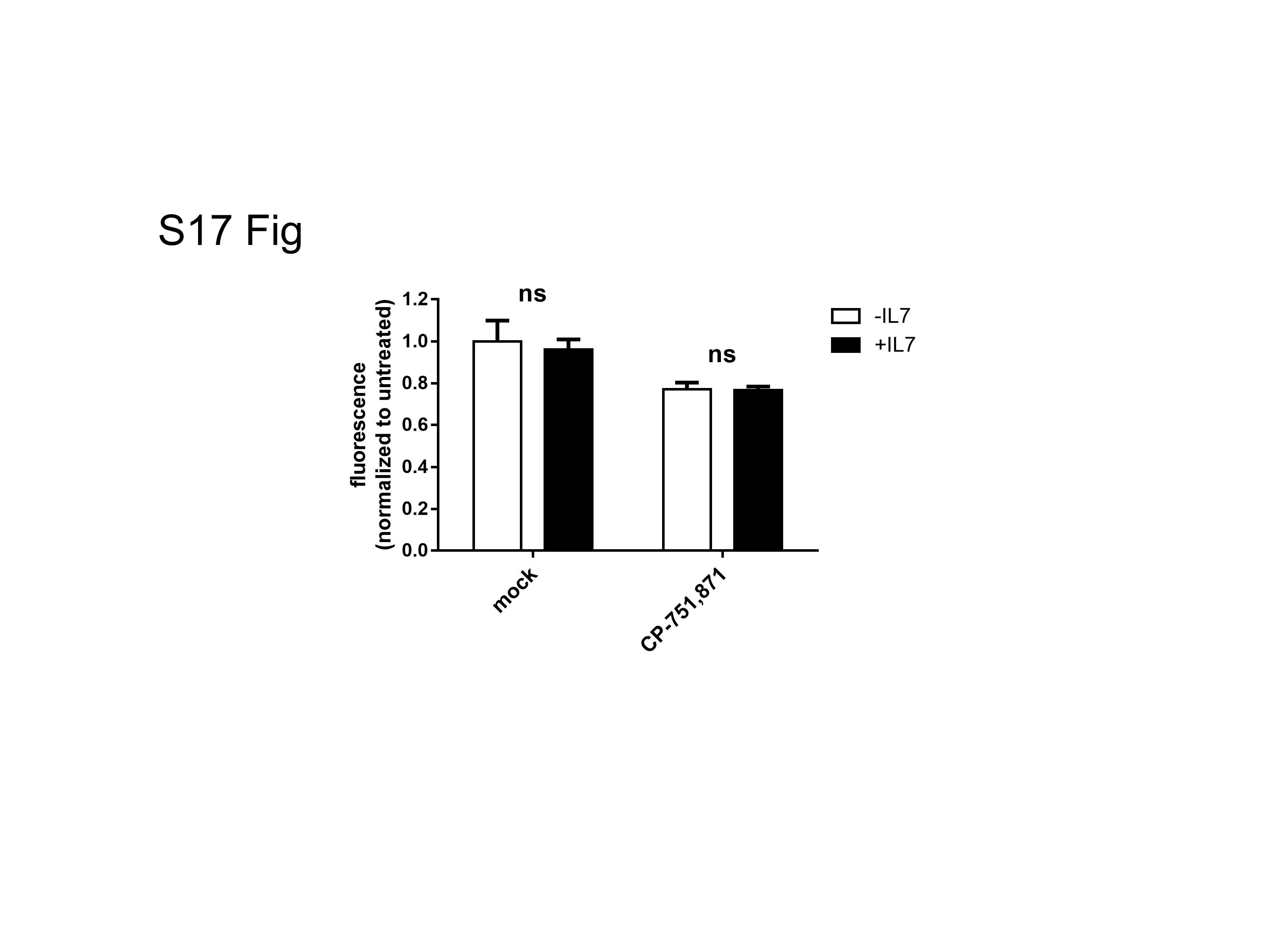

Supplement: S17 Fig — Cell growth as measured by resazurin reduction assay. HPB-ALL cells were cultured in vitro with IGF1R blocking antibody (1 μg/ml CP-751,871) with or without supplemental recombinant IL-7 (100 ng/ml) added daily for 3 days. Mean resorufin fluorescence values +/- SD after normalization to untreated control are plotted for assays performed in triplicate. ns, not significant (2-way ANOVA with Sidak’s multiple comparisons test). (TIF) [file pone.0161158.s017.TIF]

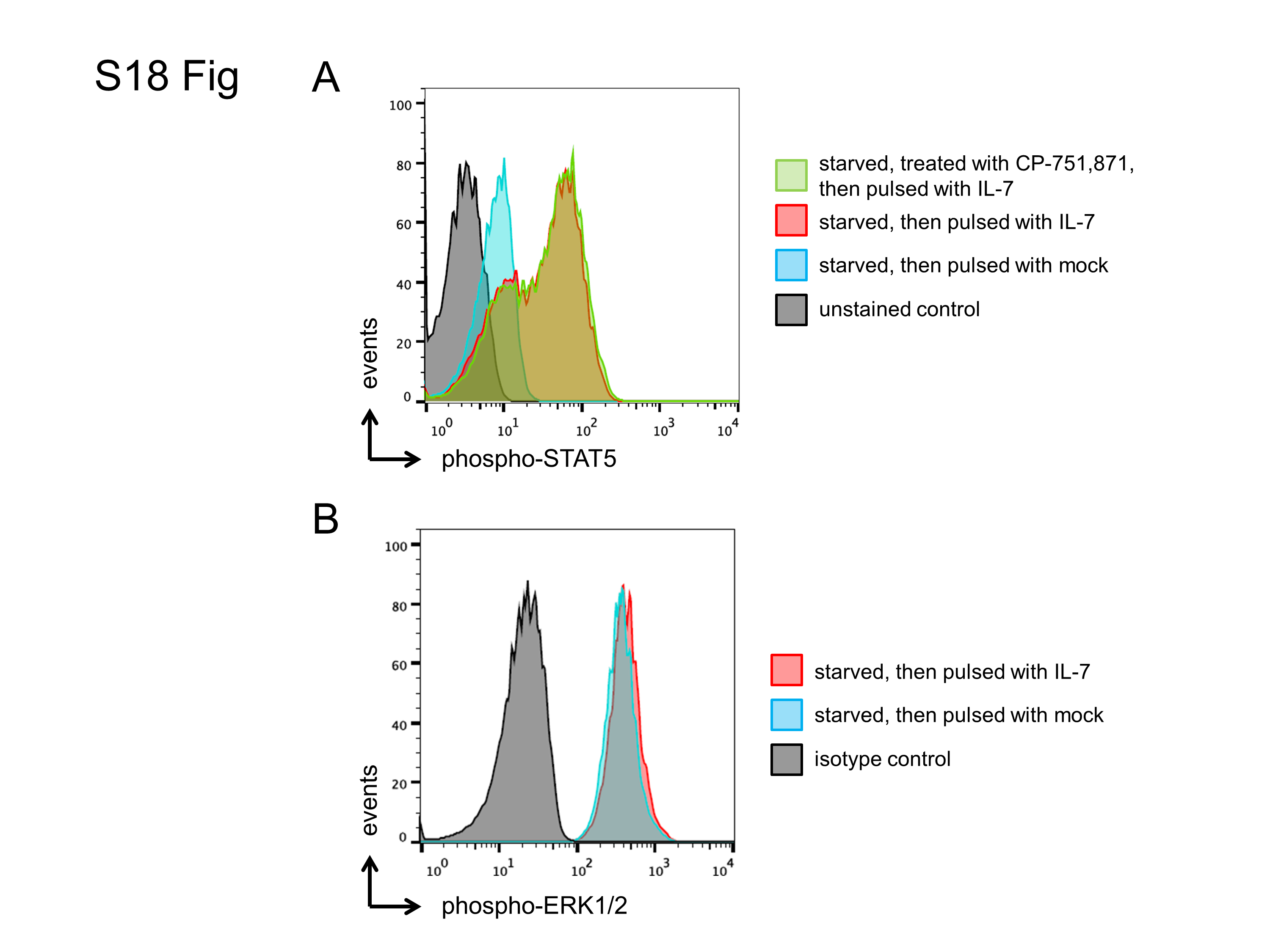

Supplement: S18 Fig — Flow cytometric analysis for intracellular phospho-STAT5 and phospho-ERK levels. HPB-ALL cells were serum starved for 24 hours, pulsed with 100 ng/ml recombinant IL-7 (Peprotech), then fixed/permeabilized, and stained with (A) anti-phospho-STAT5 (Y694) or (B) anti-phosphoERK1/2 (T202/Y204) antibodies. In (A), cells were also treated in the last hour of serum starvation (immediately prior to IL-7 pulse) with IGF1R blocking antibody (1 μg/ml CP-751,871). (TIF) [file pone.0161158.s018.TIF]
