# Supplementary material for: IGF1R Derived PI3K/AKT Signaling Maintains Growth in a Subset of Human T-Cell Acute Lymphoblastic Leukemias
Source: PLoS One. 2016 Aug 17;11(8):e0161158. doi: 10.1371/journal.pone.0161158 (PMC4988785; doi:10.1371/journal.pone.0161158)
Supplement: S2 Table — ND, not determined. (DOCX) [file pone.0161158.s020.docx]

**Table S2**

| **Cell Line** | **PTEN expression** | **PTEN reference** | **IL7R mutation status**[[1](#_ENREF_1)] |
| --- | --- | --- | --- |
| **ALL-SIL** | + | Zuurbier[[2](#_ENREF_2)] | WT |
| **HPB-ALL** | + | Zuurbier[[2](#_ENREF_2)] | WT |
| **TALL-1** | + | Zuurbier[[2](#_ENREF_2)] | WT |
| **HSB** | + | Zuurbier[[2](#_ENREF_2)] | WT |
| **KOPTK1** | + | You[[3](#_ENREF_3)] | WT |
| **SUPT1** | - | Zuurbier[[2](#_ENREF_2)] | WT |
| **CTV-1** | + | This report | ND |
| **RPMI 8402** | - | Zuurbier[[2](#_ENREF_2)] | WT |
| **MOLT4** | - | Zuurbier[[2](#_ENREF_2)] | WT |
| **SUPT13** | - | This report | WT |
| **CUTLL1** | + | You[[3](#_ENREF_3)] | ND |
| **THP6** | - | This report | ND |
| **PF-382** | - | Zuurbier[[2](#_ENREF_2)] | WT |
| **MKB1** | - | This report | ND |
| **BE13** | + | Zuurbier[[2](#_ENREF_2)] | WT |
| **MOLT13** | - | This report | ND |
| **Karpas45** | - | Zuurbier[[2](#_ENREF_2)] | ND |
| **Jurkat** | - | You[[3](#_ENREF_3)] | WT |
| **DND41** | + | Zuurbier[[2](#_ENREF_2)] | mutated |
| **Loucy** | - | Zuurbier[[2](#_ENREF_2)] | WT |
| **KE-37** | - | Zuurbier[[2](#_ENREF_2)] | WT |
| **REX** | ND |  | ND |
| **PEER** | + | Zuurbier[[2](#_ENREF_2)] | WT |
| **P12 Ichikawa** | - | Zuurbier[[2](#_ENREF_2)] | WT |
| **CCRF-CEM** | - | Zuurbier[[2](#_ENREF_2)] | WT |
| **SUPT11** | + | This report | WT |
| **MOLT16** | - | Zuurbier[[2](#_ENREF_2)] | WT |
